# The Role of Specialized Pro-Resolving Mediators in Cystic Fibrosis Airways Disease

**DOI:** 10.3389/fphar.2020.01290

**Published:** 2020-09-02

**Authors:** Maelle Briottet, Mickael Shum, Valerie Urbach

**Affiliations:** Institut national de la santé et de la recherche médicale (Inserm) U955, Institut Mondor de Recherche Biomédicale (IMRB), Créteil, France

**Keywords:** cystic fibrosis, CF airway inflammation, specialized pro-resolving lipid mediators, bioactive lipids, lipoxin, resolvin

## Abstract

Cystic Fibrosis (CF) is a recessive genetic disease due to mutations of the Cystic Fibrosis Transmembrane Conductance Regulator (*CFTR*) gene encoding the CFTR chloride channel. The ion transport abnormalities related to *CFTR* mutation generate a dehydrated airway surface liquid (ASL) layer, which is responsible for an altered mucociliary clearance, favors infections and persistent inflammation that lead to progressive lung destruction and respiratory failure. The inflammatory response is normally followed by an active resolution phase to return to tissue homeostasis, which involves specialized pro-resolving mediators (SPMs). SPMs promote resolution of inflammation, clearance of microbes, tissue regeneration and reduce pain, but do not evoke unwanted immunosuppression. The airways of CF patients showed a decreased production of SPMs even in the absence of pathogens. SPMs levels in the airway correlated with CF patients’ lung function. The prognosis for CF has greatly improved but there remains a critical need for more effective treatments that prevent excessive inflammation, lung damage, and declining pulmonary function for all CF patients. This review aims to highlight the recent understanding of CF airway inflammation and the possible impact of SPMs on functions that are altered in CF airways.

## Introduction

CF is a multisystemic disorder with the lung disease being the main cause of morbidity and mortality. It is commonly acknowledged that the reduced ASL height in CF, is responsible for an altered mucociliary clearance which favors infections and persistent inflammation, leading to progressive lung destruction and respiratory failure (Davis, 2006). However, CF airway inflammation is excessive and ineffective against pathogens, can occur very early in the development of the disease and in some cases without any sign of infection (Balough et al., 1995; Khan et al., 1995; Armstrong et al., 1997; Balázs and Mall, 2019).

While acute inflammation is protective, excessive swarming of polymorphonuclear neutrophils (PMN) amplifies inflammation with collateral tissue damage. Therefore, the inflammatory response is normally followed by an active resolution phase to return to tissue homeostasis in which among other mediators (cytokines, chemokines, immune cells), bioactive lipids might play a crucial role. Prostaglandins and leukotrienes stimulate the initiation and propagation phases of inflammation (Samuelsson et al., 1987; Funk, 2001) and the role of other lipid mediators called specialized pro-resolving mediators (SPMs) such as lipoxins, resolvins, protectins, and maresins has been demonstrated in the resolution phase (for recent review (Serhan and Levy, 2018)). SPMs promote resolution of inflammation, clearance of microbes, tissue regeneration and reduce pain, but do not evoke unwanted immunosuppression.

Abnormal SPM production or function have been related to widely occurring disorders, including CF airway disease. The airways of CF patients showed a decreased production of SPMs even in the absence of pathogens, which is consistent with other reports showing that inflammation in CF might not only be a consequence of chronic infection but could be related to intrinsic abnormalities of the inflammatory response (Muhlebach et al., 1999; Muhlebach and Noah, 2002; Karp et al., 2004; Ringholz et al., 2014; Bartlett et al., 2016; Isopi et al., 2020). The two SPMs, lipoxin A4 (LXA4) and resolvin D1 (RvD1), have the unique ability to restore the airway surface hydration, to damp the pro-inflammatory program and to fight infection in CF airways circumventing the most difficult aspects of CF pathophysiology (Karp et al., 2004; Grumbach et al., 2009; Verrière et al., 2012; Buchanan et al., 2013; Al-Alawi et al., 2014; Higgins et al., 2014; Higgins et al., 2016; Codagnone et al., 2017; Ringholz et al., 2018; Isopi et al., 2020). Furthermore, SPM levels in the airway correlate with CF patients lung function (Chiron et al., 2008; Eickmeier et al., 2017).

The prognosis for CF has greatly improved but it remains a severe and lethal disease. One promising therapy recently emerged, with small molecule correctors of mutated CFTR, which improve CFTR function and trafficking to the plasma-membrane [for recent review, see (Lopes-Pacheco, 2019)]. However, these therapies are gene mutation specific and their long-term impact on airway inflammation is still controversial (Jarosz-Griffiths et al., 2020; Volkova et al., 2020). To date there remains a critical need for more effective treatments that prevent excessive inflammation, lung damage, and declining pulmonary function for all CF patients.

This review highlights the current understanding of CF airway inflammation in the context of the recently described role of SPMs in chronic inflammation and the possible benefits of exogenous SPMs treatment in CF airways.

## Acute Inflammation and Specialized Pro-Resolving Mediators

Specialized Pro-resolving Mediators (SPMs) are a new family of lipid mediators involved in the acute inflammatory process.

Excessive inflammation is considered a common component of a vast range of chronic diseases including vascular diseases, metabolic syndrome, cancer, and neurological and airway diseases. Chronic inflammation is a pathological condition characterized by a persistent inflammatory process which ultimately leads to tissue degradation and/or remodeling (Kotas and Medzhitov, 2015; Uluçkan and Wagner, 2017). Either innate or adaptive immune responses can be involved in chronic inflammation. Chronic inflammation stems from a failure to eliminate the excessive pathogen load and/or from a dysfunctional immune system including failure to resolve inflammation (Nathan, 2002; Nathan and Ding, 2010).

In contrast, acute inflammation is a protective immune response against microbial pathogens, tissue injury or other harmful stimuli which evolved to eliminate invading organisms and to enhance tissue repair (Germolec et al., 2018). The inflammatory process is thus normally self-limited without progressing to chronic inflammation and fibrosis, leading to a return to tissue homeostasis. Resolution was considered a passive process in which the mediators involved in generating the inflammatory response would just dilute and dissipate. With identification of the family of bioactive lipid mediators SPMs, Serhan’s team provided evidence that resolution of inflammation is an active, programmed response and not simply a process of diluting chemoattractant gradients (Samuelsson et al., 1987; Brady et al., 1995; Nathan, 2002; Serhan and Levy, 2018).

**Figure 1:**
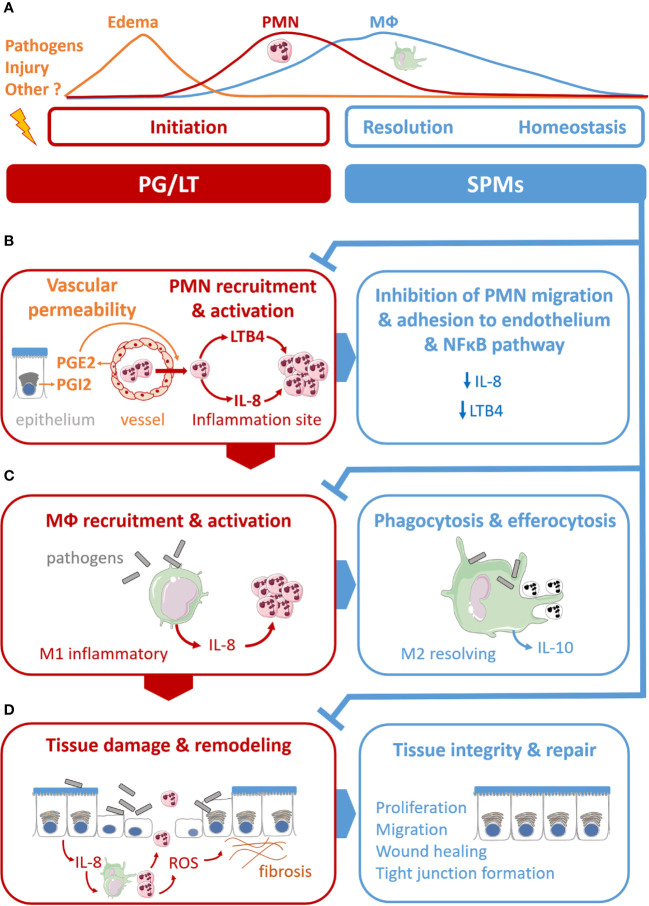
Illustration of specialized pro-resolving mediators’ (SPMs) multiple functions in the acute resolution of inflammation. **(A)** Acute inflammation evolved as a sequence of initiation and resolution phases that aim to drive back tissues toward homeostasis. The role of lipid mediators, prostaglandins (PG), leukotriene (LT), and SPMs can be related to each phase. **(B)** PGE2 and PGE2 and LTB4 play pivotal roles in the vascular response and leukocyte trafficking during the initiation phase. The SPMs, lipoxins, resolvins, protectins, and maresins produced at the inflammation, stimulate the cellular events that counter-regulate pro-inflammatory mediators and regulate polymorphonuclear (PMN) chemotaxis and migration, inhibiting LTB4 and pro-inflammatory cytokines synthesis (IL-8). **(C)** SPMs regulate monocyte and macrophage (MФ) response. SPMs drive differentiation from inflammatory MФ (M1) to resolving MФ (M2), inhibit pro-inflammatory cytokine production (IL-8) induced by bacteria, stimulate anti-inflammatory cytokines synthesis (IL-10), enhance MФ phagocytic function and efferocytosis of apoptotic PMN. **(D)** SPMs promote regeneration of tissue damaged by the inflammatory process.

Therefore, inflammatory response is currently described as a physiological process divided into initiation and resolution phases, where lipid mediators, namely prostaglandins (PG), leukotrienes (LT), and SPMs play pivotal roles (Samuelsson et al., 1980; Funk, 2001). PGE_2_ and PGI_2_ induce vascular dilatation and permeability allowing PMN trafficking from blood circulation to enter the site of inflammation (Ley et al., 2007). LTB4 plays a role as a main chemoattractant driving PMNs to the inflammatory site to phagocytose pathogens. The resolution phase, orchestrated by SPMs in concert with other mediators, starts with cessation of PMN influx (**Figures 1A, B**) (Serhan et al., 2018). The families of SPMs identified to date are lipoxins (LX), resolvins (Rv), protectins (PD), and maresins (MaR). Their roles have been described in microbial defense, pain, organ protection and tissue regeneration, wound healing, cancer, reproduction, and neurobiology-cognition. SPMs play a crucial role in inhibiting the nuclear factor κB (NF-κB) (Chiang et al., 2006; Liao et al., 2012) and the synthesis of pro-inflammatory cytokines (Schwab et al., 2007; Krishnamoorthy et al., 2010; Chiang et al., 2012). SPMs also inhibit leukocyte chemotaxis and migration (Lee et al., 1989; Soyombo et al., 1994; Serhan et al., 1995; Papayianni et al., 1996; Sun et al., 2007; Isobe et al., 2012) (**Figures 1A, B**) as well as enhance innate microbial killing and clearance by stimulating leukocytes’ phagocytosis of bacteria (Chiang et al., 2012; Chiang et al., 2015; Codagnone et al., 2018). They enhance macrophages’ efferocytosis of apoptotic immune cells and cell debris (Godson et al., 2000; Schwab et al., 2007; Dalli and Serhan, 2012; Rogerio et al., 2012; Grabiec and Hussell, 2016) (**Figure 1C**). The repair of tissue damages generated by the inflammatory process also involves SPMs in promoting tissue regeneration (Dalli et al., 2014; Dalli et al., 2016) (**Figure 1D**). Beyond innate phagocyte responses to resolve acute inflammation, SPMs appear to play critical roles in regulating adaptive immunity (Barnig et al., 2013; Krishnamoorthy et al., 2015; Chiurchiù et al., 2016).

SPMs exert their multiple actions through different cellular pathways involving diverse receptors (Chiang and Serhan, 2017). The first SPM receptor to be described is the N-formyl peptide receptor 2 (FPR2), a G protein-coupled receptor (GPCR) expressed in PMNs (Fiore et al., 1992) and other leukocytes (Chiang et al., 2006) as well as epithelial cells with high affinity for LXA4 (Fiore et al., 1994; Maddox et al., 1997; Chiang et al., 2003). Besides LXA4 and Aspirin-triggered 15-epi-LXA4 (AT-LXA4), FPR2 is also activated by Annexin A1 (ANXA1) (Perretti et al., 2002a; Bena et al., 2012). To date, five other specific SPM GPCRs have been identified in human and murine cells: GPR32, ChemR23, GPR18, GPR37, and GPR148. FPR2 and GPR32 mediate the RvD1, RvD3, and RvD5 immuno resolving actions (Krishnamoorthy et al., 2010; Hellmann et al., 2011; Recchiuti et al., 2011; Chiang et al., 2012; Gavins et al., 2012; Barnig et al., 2013; Buchanan et al., 2013; Dalli et al., 2013b; Lee and Surh, 2013; Recchiuti et al., 2014; Norling et al., 2016). Human GPR32 is mostly expressed in PMNs, monocytes, macrophages, and endothelial cells (Krishnamoorthy et al., 2010). Of note, RvD1 contributes to resolving inflammation by regulating a set of microRNAs specific to each receptor, FPR2 and GPR32 (Krishnamoorthy et al., 2010; Recchiuti et al., 2011). RvE1 and RvE2 exert potent and cell-specific actions on leukocytes *via* Chem32 (Tjonahen et al., 2006; Oh et al., 2012). GPR18 has been identified as the receptor for RvD2 (Chiang et al., 2015; Chiang et al., 2017), GPR37 for PD1 (Bang et al., 2018), and GPR148 for MaR1 (Chiang et al., 2019).

The cell signaling pathways activated by SPMs appear to be cell specific and do not depend solely on the receptor they bind to. In human epithelial cells, LXA4, and Mar1 stimulate a large increase in intracellular calcium promoting ASL height increase and epithelial repair in airway epithelia, and mucin secretion in conjunctival goblet cells (Lee et al., 1989; Nigam et al., 1990; Romano et al., 1996; Bonnans et al., 2003; Grumbach et al., 2009; Buchanan et al., 2013; Higgins et al., 2014; Hodges et al., 2017; Ringholz et al., 2018; Olsen et al., 2020). In contrast, in PMNs, LXA4 only induces a small increase in calcium and inhibits intracellular calcium mobilization induced by LTB4 in a context of PMN migration (Lee et al., 1989; Nigam et al., 1990; Romano et al., 1996; Grumbach et al., 2009; Buchanan et al., 2013; Higgins et al., 2014; Hodges et al., 2017; Ringholz et al., 2018). In human PMNs, stimulation of the LXA4 receptor inhibits phospholipase D (PLD) activity and superoxide anion generation (Levy et al., 1999; Levy and Serhan, 2000; Levy et al., 2005b), while in rat conjunctival goblet cells, LXA4 stimulates PLD activity in order to increase mucin secretion (Hodges et al., 2017). Binding to FPR2 can mediate both pro-inflammatory or anti-inflammatory responses depending on its ligand. In PMNs, while its ligand, serum amyloid A (SAA) induces a pro-inflammatory response with increased IL-8 secretion, LXA4 triggers the resolution characterized by a decreased IL-8 synthesis (He et al., 2003).

A distinct category of SPMs is the aspirin-triggered (AT) SPMs, which have a close but distinct structure. The AT-LXA4 and AT-LXB4 (15-epi-LXB4) differ only in the *S* and *R* chirality of their 15-hydroxyl residue. Theses endogenous SPMs' biosynthesis is induced by acetylsalicylic acid (ASA, or aspirin). To date, AT-LXs, AT-Rvs of the D series, and AT-PDs have been identified (Clària and Serhan, 1995; Chiang and Serhan, 2004; Serhan et al., 2004). They exert similar pro-resolving properties (Eickmeier et al., 2013; Wang et al., 2017; Liu et al., 2018; Hu et al., 2019).

Other mediators have been shown to play a role in the resolution of inflammation [for recent reviews, see (Perretti and D’Acquisto, 2009; Wallace et al., 2015; Schett and Neurath, 2018)]. ANXA1 and its derived peptides display a wide range of pro-resolving actions, among which inhibition of neutrophil recruitment, neutrophil/endothelium interaction and efferocytosis enhancement (Perretti et al., 2002b; Hayhoe et al., 2006; Scannell et al., 2007; D’Acquisto et al., 2008; Dalli et al., 2013a). In macrophages, the purine nucleoside adenosine promotes macrophage polarization toward resolution phenotype (M2), inhibits production of pro-inflammatory cytokines, and increases VEGF (Pinhal-Enfield et al., 2003; Csóka et al., 2012). Gaseous mediators such as carbon monoxide (CO), hydrogen sulfide (H_2_S), and nitric oxide (NO) also display anti-inflammatory and pro-resolving properties. Among them, NO possesses pro-apoptotic qualities, H2S reduces leukocyte-endothelium interaction through activation of K_ATP_ channels, CO inhibits NF-κB activation and proinflammatory cytokines secretion in colonic epithelial cells. However, NO and adenosine, can also display pro-inflammatory effects depending on the concentration, localization, receptors involved, and timing in the inflammatory response [reviewed in (Brüne, 2005; Vass and Horvath, 2008)]. In addition, these mediators share common anti-inflammatory pathways with SPM signaling pathways. The ANXA1’s anti-inflammatory actions rely on its ligation to the ALX/FPR2 receptor (Perretti et al., 2002b). Adenosine mediates Cl^-^ secretion in epithelial cells through intracellular calcium signaling in CF airway epithelium (Chao et al., 1994). The pro-resolution neuromodulator netrin-1 contributes to SPM production in mice peritoneal inflammation (Mirakaj et al., 2014). CO stimulates SPM biosynthesis through the regulation of 15LO1 *in vivo* in mice and baboons as well as *in vitro* in human cells (Chiang et al., 2013; Dalli et al., 2015). Physiologic hypoxia also stimulates SPMs synthesis by M2 macrophages-neutrophils interaction (Norris et al., 2019).

In humans, SPMs which are rapidly metabolized and degraded are found at pico to nanogram levels in blood, breastmilk, the airways (breath condensate and sputum), urine, and tears (Gangemi et al., 2003; Levy et al., 2007; Psychogios et al., 2011; Mas et al., 2012; Weiss et al., 2013; Colas et al., 2014; Ringholz et al., 2014; Sasaki et al., 2015; Barden et al., 2016; Dalli et al., 2017; Eickmeier et al., 2017; English et al., 2017). The short half-life of SPMs, whether they are endogenous or synthetic, led to the manufacturing of the first analogs of LXA4 by Serhan and his collaborators have made it easier to study their biological effects. These analogs differ structurally to prevent their metabolization and thus have the advantage of being more stable (Serhan et al., 1995). Other generations of synthetic stable analogs have since been developed and have shown similar pro-resolving effects to native SPMs, such as LXA4 analogs (Maddox et al., 1997; Mitchell et al., 2002), RvD1 analog (Orr et al., 2015), and RvE1 analog (Arita et al., 2006).

SPMs levels are significantly increased in inflammatory exudates in rheumatoid arthritis, skin blisters, bronchoalveolar lavage (BAL) fluids from patients with airway diseases (Lee et al., 1990; Karp et al., 2004; Planagumà et al., 2008; Morris et al., 2009; Ringholz et al., 2014; Norling et al., 2016; Motwani et al., 2018a; Motwani et al., 2018b). However, SPMs levels are shown to be decreased and unbalanced compared to pro-inflammatory eicosanoids in patients with chronic conditions including airway diseases. Lower LXA4 and LXB4 levels in sputum, BAL, and blood correlate with a more severe asthma phenotype. LXA4 levels have been found to be reduced in the sputum of Chronic Obstructive Pulmonary Disease (COPD) patients during exacerbation (Levy et al., 2005a; Vachier et al., 2005; Levy et al., 2007, 1; Schwab et al., 2007; Planagumà et al., 2008; Balode et al., 2012; Liao et al., 2012; Karra et al., 2015; Codagnone et al., 2018; Serhan et al., 2018). Altogether, SPMs, which are conserved structures, are now strong candidates for the role of main actors of the resolution phase of acute inflammation. Reports of decreased SPM biosynthesis or function in inflammatory chronic diseases suggest the involvement of SPMs in their pathogenesis. From these observations, additional fundamental studies aim to shed more light on the precise role of SPMs in these conditions. The next chapter will tackle the known alterations in lipid metabolism and SPMs biosynthesis in CF.

## Abnormal Lipid Metabolism and SPMs Biosynthesis in CF

Along with ANXA1 and NO, an abnormal SPMs biosynthesis was reported in CF (Karp et al., 2004; Dalli et al., 2010; Ringholz et al., 2014; Güney et al., 2019).

**Figure 2:**
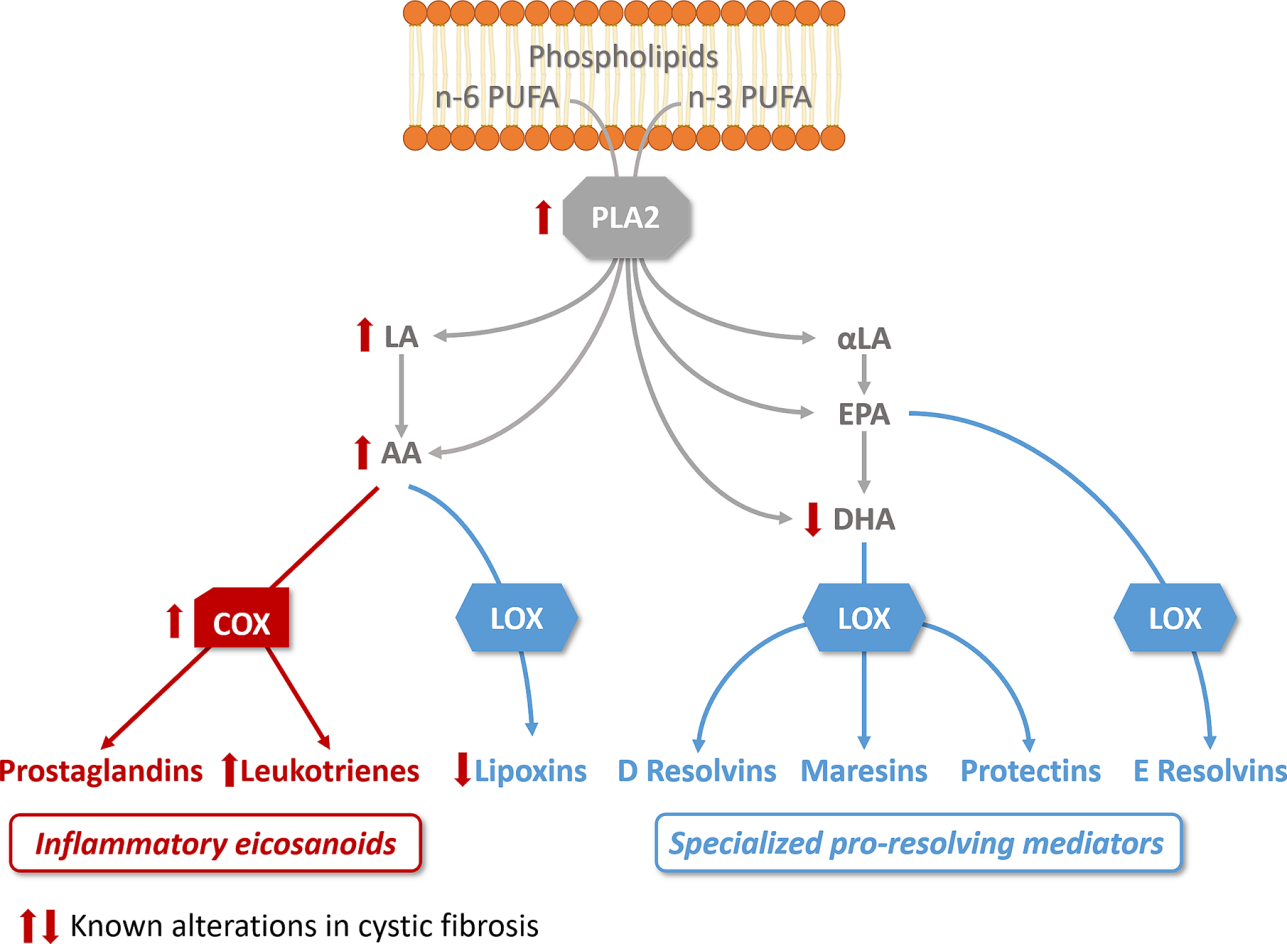
Altered lipid metabolism in cystic fibrosis. The activity of phospholipase A2 (PLA2), which releases essential fatty acid (n-6 and n-3 polyunsaturated fatty acids, PUFA) from phospholipids, is increased in CF bronchial epithelial cells. Free arachidonic acid (AA) which can be released from phospholipids or metabolized from n-6 linoleic acid (LA), is the precursor of prostaglandins and leukotrienes via cyclooxygenase activity (COX), which is increased in CF. AA is also a substrate for lipoxygenases (LOX) to produce lipoxins, found reduced in CF. The n-3 PUFA, α-linoleic acid (α-LA) is metabolized to ecosapentanoic acid (EPA) and docosahexaenoic acid (DHA), found decreased in CF. EPA and DHA are the precursors of resolvins, protectins, and maresins. The pathways involved in the biosynthesis of pro-inflammatory eicosanoids are illustrated in red, the pathways involved in the biosynthesis of specialized pro-resolving mediators in blue.

SPMs biosynthesis results from the interaction of enzymes called lipoxygenases (LOX) and cyclooxygenases (COX) to metabolize essential n-3 (omega 3) and n-6 (omega 6) polyunsaturated fatty acids (PUFA). The main PUFA substrates are arachidonic acid (AA, n-6), eicosapentaenoic acid (EPA, n-3), and docosahexaenoic acid (DHA, n-3) (Serhan et al., 1984; Samuelsson et al., 1987). Different cell types that differentially express LOX and COX enzymes cooperate by exchanging intermediates to produce the final active metabolites (Papayianni et al., 1996; Folco and Murphy, 2006; Sala et al., 2010). While free AA gives rise to the lipoxin family of SPMs, it is also the precursor of pro-inflammatory mediators PG and LT depending on which metabolic enzyme is involved (Samuelsson et al., 1987; Folco and Murphy, 2006). The DHA and EPA metabolites resolvins, protectins, and maresins exert mainly resolving actions (**Figure 2**).

**Figure 3:**
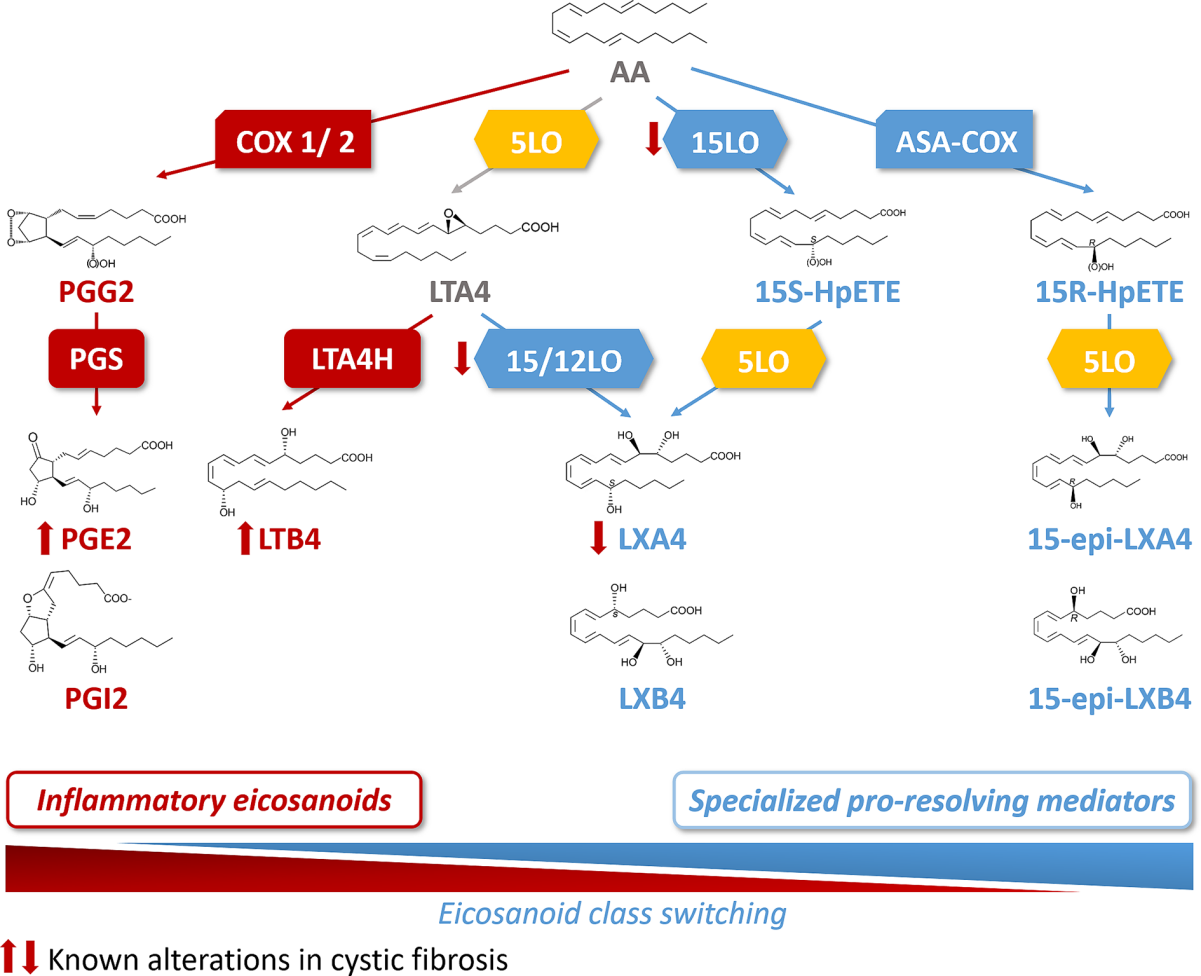
Arachidonic acid (AA) metabolome and abnormal class switching in CF. Arachidonic acid (AA) is metabolized by cyclooxygenase 1 and 2 (COX1/2) and prostaglandin synthase (PGS) into the inflammatory eicosanoids, prostaglandins (PGE2 and PGI2). AA is also a substrate for 5LO to produce leukotriene A4 (LTA4). The 15 lipoxygenase (15LO) activity drives then the SPM, lipoxin A4 (LXA4) synthesis (from LTA4) at the expense of the production of the inflammatory lipid, leukotriene B4 (LTB4) that involves leukotriene A4 hydrolase (LTA4H) activity. The sequential activities of 15LO and 5LO drive the biosynthesis of LXA4, with an intermediate product, 15S hydroperoxyeicosatetraenoic (15S-HpETE). Acetylsalicylic acid inhibited COX (ASA-COX) leads to the production of 15R hydroperoxyeicosatetraenoic acid (15-R-HpETE) which is converted by the 5LO to the SPM, 15-epi-LXA4. In CF, the abnormal class switching from inflammatory biosynthetic pathway to the pro-resolving pathway results into an increased LTB4 and decreased LXA4 biosynthesis in the airway which are responsible for the sustained inflammation.

Acute inflammation is the result of a temporal eicosanoids class switching where COX-derived prostaglandins precede biosynthesis of lipoxins. This eicosanoids class switching, characterized by an increased biosynthesis of SPMs and a decrease of eicosanoids involved in the initiation of inflammation, results from the coordinated action of enzymes such as the different isoforms of lipoxygenases (15LO, 5LO, and 12LO), of COX (COX1 and COX2), as well as the leukotriene A4 hydrolase (LT4H) (**Figure 3**). These enzymes are selectively expressed in the cell types involved in the inflammatory process. The 15LO expressed in airway epithelial cells and macrophages and the 12LO in platelets play a central role in the class-switching from the pro-inflammatory lipid mediator LTB4 toward SPMs. Isolated PMNs exposed to PGE2 (to mimic exudates) switched eicosanoid biosynthesis from predominantly LTB4 and 5LO-initiated pathways to LXA4, which is a 15LO product that stops PMN infiltration (Levy et al., 2001). Indeed, the activity of 15LO favors LXA4 synthesis at the expense of LTB4 synthesis that involves LTA4H activity (Haeggström and Funk, 2011) (**Figure 3**). The cellular location of 5LO also plays a crucial role in the LTB4/SPM ratio. Inhibition of the CaM kinases by RvD1 favors the extra-nuclear location of 5LO, and the LXA4 biosynthesis at the expense of LTB4 (Fredman et al., 2014). Finally, while COX1 and COX2 metabolize AA into the inflammatory eicosanoids PGE2 and PGI2, acetylsalicylic acid (ASA) inhibits COX1 and modifies COX2 by acetylation (ASA-COX), leading to a shift from production of the precursor of PG, to 15-R-HpETE which is converted by the 5LO to AT-LXA4 (15-epi-LXA4) (Birnbaum et al., 2007) (**Figure 3**).

Before the gene defect responsible for CF was identified, it was suggested that fatty acid metabolism abnormalities were responsible for the clinical symptoms of the CF disease and this has been confirmed in numerous further studies (Kuo et al., 1962) [for recent review, (Wheelock and Strandvik, 2020)]. Lower levels of palmitic, stearic, and linoleic acid (LA), were reported in plasma of CF patients compared to healthy controls (Hubbard et al., 1977; Gibson et al., 1986; Thompson, 1989; Roulet et al., 1997; Strandvik et al., 2001; Al-Turkmani et al., 2007). Initially thought as the result of malabsorption by CF patients (Hubbard et al., 1977), an abnormal lipid metabolism related to CFTR dysfunction was further demonstrated. An imbalance of PUFAs with increased release of AA and decreased levels of DHA in CF was established (Underwood et al., 1972; Bhura-Bandali et al., 2000; Freedman et al., 2004) (**Figure 2**). However, DHA supplementation in CF patients, in order to correct the imbalance between AA and DHA gave a controversial outcome (Lloyd-Still et al., 2006; Coste et al., 2007; Oliver and Watson, 2016; Teopompi et al., 2019). The direct link between the altered fatty acid levels and CFTR dysfunction is unclear. The activity of phospholipase A2 (PLA2), which releases AA from the membrane phospholipids and can interact with CFTR, is increased in human CF bronchial epithelial cells and in CFTR-knockout mice (Miele et al., 1997; Six and Dennis, 2000; Ghosh et al., 2006; Borot et al., 2009; Dif et al., 2010). Free AA is led down the PG metabolic pathway by the COX enzymes, which are upregulated in sinonasal mucosa of CF patients (Roca-Ferrer et al., 2006; Owens et al., 2008) (**Figure 2**). Consistent with this, polymorphisms downregulating COX1 and COX2 expression in CF patients are associated with a better clinical outcome (Czerska et al., 2010). The first studies focused on LXA4 levels and LXA4 to PMN ratio measured in BAL fluids of CF subjects compared to non-CF subjects and suggested a relative decrease of LXA4 to PMN concentrations (Karp et al., 2004). In contrast, Starosta et al. observed no differences in the absolute LXA4 BAL fluids concentrations between CF patients and controls with a similar degree of neutrophilic airway inflammation. Concentrations were also similar in CF patients with mild versus more severe airway inflammation (Starosta et al., 2006). Another study showed that CFTR defects dampen LXA4 biosynthesis during platelets-PMN interaction (Mattoscio et al., 2010). The ratio of SPMs to the pro-inflammatory eicosanoid LTB4 has also been evaluated by Ringholz et al. in BAL fluid samples from children with CF. An imbalance between resolving (LXA4) and pro-inflammatory eicosanoids (LTB4) was found, suggesting a defect in eicosanoid class switching (Ringholz et al., 2014). Furthermore, a reduced expression of the 12/15LO in the airway of a CF mice model as well as a reduced 15LO2 isoform level in the BAL fluid of patients with F508del mutation (Karp et al., 2004; Ringholz et al., 2014). Similarly, 15LO2 expression has been shown to be decreased by 50% in the nasal polyps of CF patients compared to non-CF (Jeanson et al., 2014) (**Figure 3**). A defective activity of 12LO, another lipoxygenase expressed in platelets, has been shown when inhibiting CFTR (Mattoscio et al., 2010). Although the cellular mechanism by which CFTR could affect LOX levels and/or activities remains unclear, these data are consistent with an abnormal SPMs biosynthesis in CF.

Taken together, the altered lipid metabolism that could result in an imbalance of eicosanoids with higher pro-inflammatory mediators as compared to SPMs would be coherent with the observations of the sustained and inefficient CF airway inflammation as detailed below.

## Inflammation in CF Airway

CF is a recessive monogenic disease due to mutations in the Cystic Fibrosis Transmembrane conductance Regulator (*CFTR*) gene (Kerem et al., 1989; Riordan et al., 1989; Rommens et al., 1989). Over 2,000 mutations of *CFTR* are known, although around two thirds of patients have at least one copy of the F508del mutation (Cystic Fibrosis Mutation Database; Bobadilla et al., 2002). While CFTR dysfunction may affect many organs, the inflammatory airway disease leading to progressive lung damage is the main cause of morbidity and mortality of CF patients (Davis, 2006). Multiple pathways involved in CF airway inflammation have been described.

### Ion Transport and Airway Surface Liquid Abnormalities

The dehydrated airway surface liquid (ASL) resulting from ion transport abnormalities is a critical feature of the CF airway pathogenesis (Boucher, 2007). The main CFTR function is to transport chloride from the cytosol to the lumen (Anderson et al., 1991; Linsdell, 2017). CFTR also regulates other epithelial functions (Stutts et al., 1995; Greger et al., 1996), including the activity of the sodium channel (ENaC) located in the same membrane (Hanukoglu and Hanukoglu, 2016). Although several reports suggested no change in ENaC activity associated with CFTR dysfunction (Chen et al., 2010; Itani et al., 2011; Collawn et al., 2012; Fisher et al., 2013; Sun et al., 2014; Tuggle et al., 2014), other functional studies have brought evidence supporting CFTR role in inhibiting sodium absorption by ENaC (Kunzelmann et al., 1995; Ismailov et al., 1996; Kunzelmann et al., 2000; König et al., 2001; Kunzelmann, 2003; Gentzsch et al., 2010; Lazrak et al., 2011). At the expression level, while the α- and β-ENaC subunit amount was reported to be increased in CF nasal epithelium the γ-ENaC was decreased (Bangel et al., 2008). Consistent with a role for ENaC in CF, similar features of human CF lung disease have been reproduced when ENaC is overexpressed in mice (Mall et al., 2004).

Both transepithelial chloride secretion involving chloride channels such as CFTR and the transepithelial sodium absorption *via* ENaC, finely regulate cellular and paracellular water movements generating the ASL layer (Tarran et al., 2001; Tarran, 2004). The ASL constitutes the first line of innate defense in controlling the mucociliary clearance process, trapping and neutralizing inhaled foreign particles in the mucus layer that are then removed from the airways by the ciliated cells (Knowles and Boucher, 2002; Button et al., 2012; Shei et al., 2018). In CF, ion transport abnormalities lead to a reduced ASL height (Boucher, 2007) and result in an impaired ciliary beating, favors mucus plugging in the airways, which constitutes a viscous and nutritive medium for pathogens proliferation in the respiratory tract, especially bacteria.

The content of the ASL layer of CF patients is also affected by dysregulation of bicarbonate transport. Firstly observed in pancreas, decreased bicarbonate secretion has been related to abnormal chloride secretion (Marino et al., 1991). CFTR has been shown to regulate bicarbonate secretion; either directly by being permeant (Poulsen et al., 1994; Illek et al., 1997; Kim et al., 2014), or by interacting with other transporters, such as the chloride/bicarbonate exchangers, SLC26A4 also named pendrin, and/or the ATP12A, a non-gastric form of H^+^/K^+^-ATPase expressed at the apical side of airway epithelia (Garnett et al., 2011; Scudieri et al., 2018). Indeed, H^+^-K^+^-ATPase and CFTR-dependent bicarbonate secretion regulate extracellular pH (Coakley et al., 2003). The lack of bicarbonate secretion due to altered CFTR activity or ATP12A increased expression in CF human airways secondary to inflammation and infection are consistent with a more acidic ASL pH in CF (Song et al., 2006; Cho et al., 2011; Scudieri et al., 2018). Furthermore, ASL acidification in CF bronchial epithelial cells can be driven by hyperglycemia and *P. aeruginosa*-related lactate secretion which monitors H^+^ secretion (Garnett et al., 2016). Although ASL pH is difficult to measure and ASL pH decrease in CF is still controverted (McShane et al., 2003; Schultz et al., 2017), more acidic ASL could explain some aspect of the pathogenesis of the disease. Indeed, an acidified environment is beneficial for bacteria survival. *In vivo* studies on CF newborn pigs and *in vitro* data obtained on human airway epithelial cells have demonstrated that bacterial killing is impaired in CF due to more acidic ASL pH (Pezzulo et al., 2012; Simonin et al., 2019).

### Chronic Airway Infection and Inflammation

In CF, the lung function decline correlates with the chronic colonization by pathogens, including the bacteria *Pseudomonas aeruginosa* (Nixon et al., 2001; Emerson et al., 2002; Konstan et al., 2007) which are potent triggers of PMN response. However, in CF airways challenged by bacteria or viruses, inflammation appears disproportionate to the degree of infection, with a high PMN infiltration and release of pro-inflammatory molecules, such as TNF-α, IL-8, IL-6, IL-1β, proteases, oxidants, PG, and LT, together leading to bronchiectasis and fibrosis (Konstan et al., 1993; Balough et al., 1995; Bonfield et al., 1995; Khan et al., 1995; Noah et al., 1997; Muhlebach et al., 1999; Tirouvanziam et al., 2000; Muhlebach and Noah, 2002). As the first line of defense against microbial agents, macrophages can induce inflammation by stimulating the immune system but they also contribute to ending the inflammation process and to the return to tissue homeostasis, by clearing microbes and dead PMNs for instance. These polar roles define two macrophage subtypes with distinct gene signatures, a pro-inflammatory subtype (M1) and a pro-resolving one (M2) (Mills et al., 2000). M1 cells produce high levels of the pro-inflammatory TNFα, IL-1β, IL-6, and IL-12, while M2 cells secrete high levels of immune-resolving IL-10 and TGFβ1 compared with M1 cells (Bystrom et al., 2008). It has been suggested that altered macrophage function contributes to the sustained inflammation in CF airways (Bruscia and Bonfield, 2016). CF macrophages are hyper-responsive, producing a high amount of pro-inflammatory cytokines when exposed to bacterial stimuli (Bruscia et al., 2009; Bruscia et al., 2011) but showed a defective ability to clear bacteria (Di et al., 2006; Deriy et al., 2009; Porto et al., 2011). There have been reports of CF macrophages failing to polarize into anti-inflammatory M2 and M1 macrophages displaying a hypermetabolic state (Tarique et al., 2017; Lara-Reyna et al., 2019).

Early reports indicated signs of inflammation in the airways of young CF patients even in the absence of infection and questioned the nature of the relationship between infection and inflammation in the CF airways (Balough et al., 1995; Khan et al., 1995; Armstrong et al., 1997). Thanks to systematic newborn screening and methodical, precise phenotyping from longitudinal studies, the early lung disease onset and progression in CF has been better characterized. As early as infancy, CF patients’ lower airways show signs of mucus plugging and altered lung structure (Martínez et al., 2005; Stick et al., 2009). Furthermore, BAL fluid samples collected from infants bear evidence of active inflammation with high PMN counts and neutrophil elastase and pro-inflammatory cytokine levels, even before patients encounter their first infection by the conventional CF pathogens (Pillarisetti et al., 2011; Montgomery et al., 2017; Ranganathan et al., 2017; Balázs and Mall, 2019). The presence of sterile inflammation has also been observed in animal models, notably in CFTR-knockout ferrets that show signs of neutrophil-mediated inflammation despite being infection-free (Keiser et al., 2015; Rosen et al., 2018).

Despite the identification of the CFTR gene and the growing mutation database, establishing a correlation between CF genotype and clinical phenotype remains difficult, considering the vastly different disease courses between patients, even between twin siblings sharing the same CFTR mutations (Kerem et al., 1990; Mekus et al., 2000). Genome-wide association studies with large patient cohorts have identified genetic variants of specific loci associated with disease severity, called CF modifier genes, that contribute to phenotypic variability [see recent reviews (Lim et al., 2018; Shanthikumar et al., 2019)]. Among the proteins encoded by the CF modifier genes, some have direct protein-protein interaction with CFTR and/or are involved in immune response (Tugores et al., 2001; Wright et al., 2011; Stanke et al., 2014; Corvol et al., 2015). The trachea of CF and non-CF newborn pigs when challenged by an inflammatory stimulus revealed differential transcriptomes (Bartlett et al., 2016). Therefore, beyond ion transport abnormalities, intrinsic immune abnormalities related to CFTR dysfunction could be involved in the pathogenesis of the CF airway disease.

### Dysregulated Calcium Homeostasis

Calcium is a major intracellular second messenger playing a key role in immune functions, among which cytokines secretion and PMN recruitment (Berridge et al., 2003; Immler et al., 2018). Calcium homeostasis has long been known to be dysregulated in CF (Katz et al., 1984; Cabrini and De Togni, 1985).

Initial studies supported the hypothesis of an acquired response from the airway epithelial cells to exogenous pathogens such as *P. aeruginosa* leading to calcium mobilization and hyperinflammation (Denning et al., 1998; Hybiske et al., 2007). Further studies demonstrated that the endoplasmic reticulum (ER), a major intracellular calcium storage compartment, undergoes stress and expansion in CF airway epithelial cells as the result of chronic infection and inflammation (Ribeiro et al., 2005a; Ribeiro et al., 2005b; Ribeiro, 2006). Other studies have suggested that ER stress, NF-κB activation, and IL-8 related expressions are closely linked to CFTR channel mistrafficking and its retention in the ER of different CF models (Weber et al., 2001; Knorre et al., 2002; Antigny et al., 2008a). The ER stress normally results in unfolded protein response (UPR) mediated by proteins located in the ER membrane. However, atypical UPR activation fails to resolve the ER stress in CF and sensitizes the innate immune system to respond more vigorously to microbial challenge (Ribeiro et al., 2005b; Ribeiro, 2006; Kerbiriou et al., 2007; Lin et al., 2008; Blohmke et al., 2012). Finally, calcium transporters are also reported to be altered in CF. In the ER, the retention of the F508del CFTR protein leads to impaired activity of SERCA pump and IP3 receptor, altering calcium exchange between the ER and with the cytosol (Antigny et al., 2008a; Antigny et al., 2008b; Philippe et al., 2015). Calcium influx and efflux at the plasma-membrane have been shown to be impaired by dysfunctional coupling of mutated CFTR with transient receptor potential canonical channels 6 (TRPC6), altered plasma-membrane calcium pump and upregulated complex Orai1/STIM1 formation that increases IL-8 secretion (Antigny et al., 2011; Balghi et al., 2011; Philippe et al., 2015).

### Mucus

Mucus was for long observed to be more viscous and thick, in CF airways in *in vitro* and *in situ* studies (Derichs et al., 2011). In addition to altered ion and fluid transport that result in a dehydrated mucous layer, the CF airways are characterized by goblet cell and glandular hyperplasia and subsequent overproduction of the two secreted mucins MUC5B and MUC5AC, which has been reported in both CF patients and CF model systems (Henderson et al., 2014; Esther et al., 2019). Furthermore, structural mucus abnormalities have been described in CF. Analysis of airway mucins from newborn CF pigs’ freshly excised airways revealed that MUC5B strands remained attached to submucosal glands and MUC5AC, secreted by goblet cells, is more present and forms sheets that cover MUC5B strands (Ostedgaard et al., 2017). The early work of Inglis, Ballard et al. on excised porcine distal bronchi enlightened the role of chloride and water transport on mucus rheological properties (Inglis et al., 1997; Inglis et al., 1998; Inglis et al., 1999; Ballard et al., 2002). In addition, Perez-Vilar suggested that lower pH would favor interchain disulfide bonds and the ASL volume depletion may increase MUC5B and MUC5AC concentrations and favor interactions (Perez-Vilar and Boucher, 2004). Other studies suggested that mucus swelling and hydration is driven by the Donnan effects (unbalanced repartition of charged particles over a porous barrier) and that HCO_3_^-^ plays a key role in the swelling of the mucins favoring a decrease content of calcium associated mucins (Quinton, 2008; Chen et al., 2010). From the new-born CF piglets and human bronchial epithelium studies, the impaired mucocilliary clearance and rheological properties of mucus appeared dependent on both chloride and pH (Hoegger et al., 2014; Gorrieri et al., 2016; Hill et al., 2018; Hughes et al., 2019). Finally, a pilot study realized on 12 CF volunteers supported that nebulized NaHCO_3_ was safe and well tolerated and it permit to increase ASL pH and change the rheology of the sputum (Gomez et al., 2020).

### Oxidative Stress

Oxidative stress which arises from the loss of the oxidant/antioxidant balance shifted toward oxidant production is described in many airway diseases including CF and triggers lung injuries (Brown et al., 1996) and pro-inflammatory mechanisms (Brown et al., 1996; MacNee, 2001; Boncoeur et al., 2008; Chen et al., 2008; Bartling and Drumm, 2009; Park et al., 2009; Kelly-Aubert et al., 2011; Pongnimitprasert et al., 2012). Increased levels of reactive oxygen species (ROS), such as superoxide anion, and involvement of its main producer, the NADPH oxidase (NOX) enzyme, have been demonstrated in CF mice models and human cell lines (Esposito et al., 1999; Velsor et al., 2006; Pongnimitprasert et al., 2012). In CF airway epithelial cells, the morphology and functions of mitochondria, the main cellular component involved in ROS production (Favia et al., 2019) are altered (Feigal et al., 1982; Von Ruecker et al., 1984; Antigny et al., 2009) and restored by correction of F508del CFTR mutation (Valdivieso et al., 2007; Taminelli et al., 2008; Valdivieso et al., 2012; Atlante et al., 2016). Furthermore, *P. aeruginosa* infection and inflammation-related production of ROS by PMNs and macrophages exacerbate alterations of mitochondrial functions, DNA, and morphology in normal lung epithelial cells. All of these mitochondrial alterations contribute to the establishment of oxidative stress in the airways (Maurice et al., 2019; Causer et al., 2020). Studies from CF mice model, human cell lines, or BAL fluids have shown a reduced uptake, synthesis or activity of antioxidant defenses, such as superoxide dismutase, peroxidases, catalases, and reduced glutathione (GSH) which further enhances oxidative stress (Farrell et al., 1977; Roum et al., 1993; Velsor et al., 2006; Schwarzer et al., 2007; Rottner et al., 2011). In addition to CFTR permeability for GSH, polymorphisms in regulatory genes of the metabolic pathway of GSH were associated with different phenotypes of CF and its severity (Linsdell and Hanrahan, 1998; Hudson, 2001; Jungas et al., 2002; Marson et al., 2013; Marson et al., 2014). Finally, low levels of GSH in BAL could also result from its oxidation by pro-inflammatory products, such as hypochlorous acid, coming from infection-activated PMNs (Kettle et al., 2014).

### Abnormal Epithelial Repair

In CF, impaired mucociliary clearance, long-term infection, ineffective inflammation, increased ROS secretion and PMN products contribute to epithelial injury, amplifying airway inflammation (Chmiel and Davis, 2003). Altered CF airway epithelia repair has been more directly related to CFTR dysfunction and expression (Schiller et al., 2010; Itokazu et al., 2014). While CFTR pharmacologic inhibition, repression or mutation affect epithelial cell proliferation and migration and wound healing, CFTR rescue with transfection or CFTR modulators (correctors, potentiators) improves epithelial repair in airway epithelial cultures from patients with the most common mutations (Trinh et al., 2012; Dong et al., 2015; Adam et al., 2018). Other ion channels are also involved in the altered repair processes in CF. Indeed, epidermal growth factor (EGF) and its receptor EGFR signaling, along with potassium channel function are impaired in CF and contribute to reduced cell migration and proliferation (Trinh et al., 2008). The activity and expression of the calcium activated chloride channel, Anoctamin 1 (ANO1), shown to be reduced in human and mice CF airway epithelial cells, could also delay CF airway epithelial cell proliferation and migration (Ruffin et al., 2013).

### Dysregulated MicroRNA

MicroRNAs have emerged as important regulators in human physiological and pathological cell processes including the immune system, providing negative feedback regulation of inflammation and ion transport. In CF, microRNA profiling studies have shown that miR155, miR145, miR223, miR494, miR99b, let-7e, miR181b, and miR125a are increased in CF airway epithelial cell lines, CF bronchial brushing samples, and macrophages, compared to non-CF controls (McKiernan and Greene, 2015; Pierdomenico et al., 2017). In contrast, miR126, miR31, miR17 expression is decreased in CF airway epithelial cells. Decreased levels of miR126 correlate with up-regulation of TOM1, a negative regulator of pro-inflammatory cytokines. Decrease of miR31 in CF airways contributes to increased pulmonary cathepsin S production that activates the epithelial sodium channel and inactivates antimicrobial proteins (McKiernan and Greene, 2015). CFTR expression is under control of miR101 (Viart et al., 2015). Therefore, dysregulated microRNAs as critical regulators of epithelial immune responses can play a role in the intrinsic abnormalities of airway inflammation in CF.

## Impact of SPMs Demonstrated in CF Models

**Figure 4:**
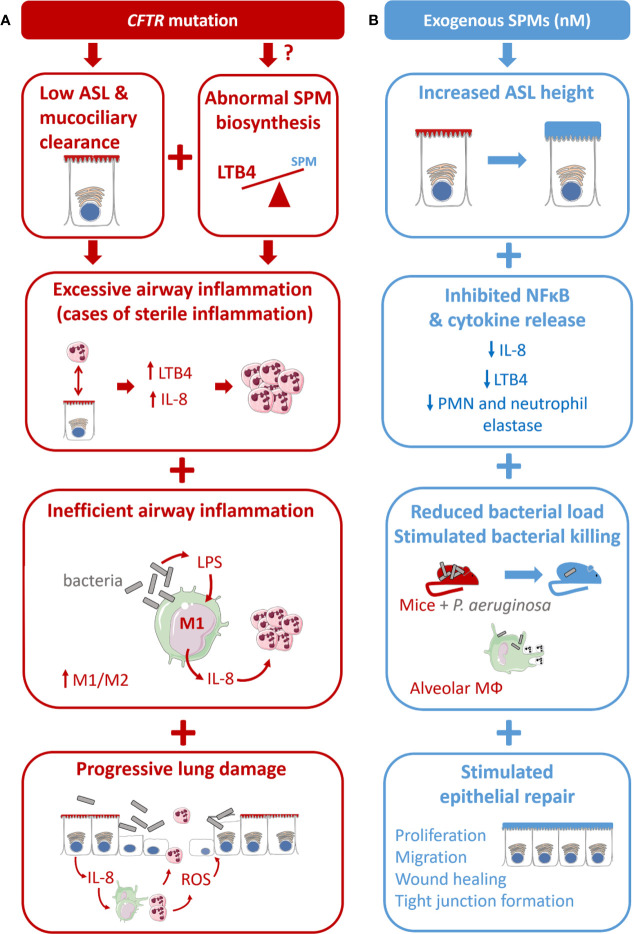
Proposed model for CF airway disease and beneficial impact of exogenous SPM exposure. **(A)** CF is due to the CFTR gene mutation leading to a reduced airway surface liquid layer (ASL) height and abnormal SPM biosynthesis. CF is characterized by an excessive airway inflammation with high amount of PMN and release of pro-inflammatory mediators (cytokines and LTB4). Inflammation in CF is inefficient to clear bacteria due to altered differentiation from M1 proinflammatory macrophages to M2 pro-resolving. Chronic inflammation and sustained inflammation, including oxidative stress (ROS) lead to progressive lung damage and respiratory failure. **(B)** The benefit of SPMs exposure at nM concentration is reported in different models of the CF airway disease. SPMs enhance the ASL height increase in CF bronchial epithelial cells. SPM treatment inhibits IL-8 release induced by TNF in CF airway epithelial cells. SPMs reduce bacterial load in mice models of P. aeruginosa infection and stimulate phagocytosis and bacterial killing in isolated CF alveolar macrophages. SPMs enhance tight junction formation and wound healing by stimulating CF airway epithelial proliferation and migration.

Abnormal epithelial ion transport, excessive and non-resolving inflammation, chronic bacterial infection, and progressive lung destruction are the main features of CF airway disease, whether they are directly or indirectly related to CFTR misexpression or malfunction. Among the multiple cellular and molecular pathways involved in CF airway pathogenesis, the abnormal SPMs biosynthesis could play a central role. Indeed, SPMs have been demonstrated to regulate several distinct dysfunctions of CF airways that result in excessive and sustained inflammation (**Table 1**, **Figure 4**). Unless specified otherwise, the SPMs used in the studies described from this point on are synthetic exogenous SPMs which have the same chemical structure and half-life as their endogenous counterpart.

**Table 1:** Bioactions of SPMs demonstrated in cystic fibrosis models.

	*In vitro* studies			
	SPMs	Models	Bioactions	References
**Inflammation & infection**	LXA4	Human CF and non-CF bronchial epithelial cells (primary, NuLi-1 and CuFi-1) + *P. aeruginosa*	Delays *P. aeruginosa* invasion and migration	Higgins et al., 2016
	LXA4	Human CF peripheral blood human macrophages	Stimulates phagocytosis of zymosan particles and *P. aeruginosa*	Pierdomenico et al., 2017
	AT-LXA4 analog	Human primary bronchial epithelial cells + Short-term *P. aeruginosa*	Inhibits IL-8 secretion	Karp et al., 2004
	RvD1	Murine RAW-264.1 macrophage, Human peripheral blood macrophage, PMN and artery pulmonary endothelial cells + Long-term *P. aeruginosa*	Reduces *P. aeruginosa* bacterial burden Reduces PMNs infiltration by decreasing ICAM-1 expression and increasing vascular permeability	Codagnone et al., 2018
		Human primary CF alveolar macrophages Human bronchial epithelial cells (CuFi-1)	Enhances phagocytosis of *P. aeruginosa* Reduces IL-8 secretion	Ringholz et al., 2018
		Human primary CF leukocytes and epithelial cells	Increases *P. aeruginosa* phagocytosis Reduces genes and protein associated to NF-kB activation and leukocyte infiltration	Isopi et al., 2020
**Ion transport & hydration**	LXA4	Human CF and non-CF bronchial epithelial cells (primary and NuLi-1, CuFi-1/3/4)	Restores ASL height by increasing calcium activated Cl^-^ secretion and inhibiting ENaC activity	Verrière et al., 2012 Higgins et al., 2014 Al-Alawi et al., 2014
	RvD1	Human CF and non-CF bronchial epithelial cells (primary and NuLi-1 and CuFi-1)	Restores ASL height	Ringholz et al., 2018
**Epithelial structure**	LXA4	Human CF and non-CF bronchial epithelial cells (primary and Nu-Li and CuFi-1) + *P. aeruginosa*	Stimulates tight junction formation and ZO-1 expression and trafficking	Higgins et al., 2016
	LXA4	Human CF and non-CF bronchial epithelial cells (primary and Nu-Li and CuFi-1)	Stimulates cell proliferation, migration and wound repair in CF and non-CF cells	Buchanan et al., 2013 Higgins et al., 2014
	***In vivo* studies**			
	**SPMs**	**Models**	**Bioactions**	**References**
**Inflammation & infection**	AT-LXA4 analog	C57BL/6 mice + Short-term *P. aeruginosa*	Reduces bacterial burden and PMN infiltration	Karp et al., 2004
	RvD1	C57Bl6/N mice + Long-term *P. aeruginosa*	Reduces *P. aeruginosa* bacterial burden Reduces PMN infiltration Reduces CCL5, CXCL10, CXCL1, IL-1β, IL-17, VEGF Reduces mucous metaplasia Increases miR-21 and miR-155(cytokine secretion) and miR-21 (pathogen recognition)	Codagnone et al., 2018
		CFTR-KO mice (FABP-CFTR)	Reduces *P. aeruginosa* bacterial burden Reduces PMN infiltration Reduces histological signs of lung pathology Increases bacterial and leukocyte clearance by macrophages Improves clinical disease score	Isopi et al., 2020
**Ion transport & hydration**	RvD1	Homozygous F508del-CFTR mice (FVB/N)	Restores nasal transepithelial potential difference in CF mice	Ringholz et al., 2018

### Epithelial Ion Transport, ASL and Calcium

Several *in vivo* and *in vitro* studies have provided evidence for SPMs’ involvement in regulating airway epithelial ionic transports and ASL layer height in models of airway epithelial diseases, including CF. In a rat model of acute lung injury (ALI), LXA4, RvD1, Mar1 enhance the expression of CFTR, ENaC (ENaC-α and ENaC-γ subunits), Na,K-ATPase (β1 subunit), and aquaporins leading to the stimulation of alveolar fluid clearance (AFC) (Yang et al., 2013; Wang Q. et al., 2014; Zhang et al., 2017) (**Table 2**).

**Table 2:** Bioactions of SPMs on ion transport and mucus secretion, demonstrated in non-CF models.

Ion transport and hydration				
Conditions	SPMs	Models	Bioactions	References
***In vitro* studies**				
**Other**	LXA4	Human bronchial epithelial cells (primary and 16HBE14o-)	Stimulates Ca^2+^ -dependent Cl^-^ secretion	Bonnans et al., 2003
**Acute lung injury**	LXA4	Rat primary alveolar type II cells	Stimulates expression of CFTR	Yang et al., 2013
	Mar1		Stimulates expression of ENaC α, β and Na-K-ATPase α1, β1	Zhang et al., 2017
***In vivo* studies**				
**Acute lung injury**	LXA4	Sprague-Dawley rat	Stimulate airway fluid clearance	Yang et al., 2013
	RvD1			Wang Q. et al., 2014
	Mar1			Zhang et al., 2017
**Mucus secretion**				
**Conditions**	**SPMs**	**Models**	**Bioactions**	**References**
***In vitro* studies**				
**Other**	LXA4	Sprague-Dawley Rat primary conjunctival goblet cells	Increases glycoconjugate secretion	Hodges et al., 2017
	RvD1			Lippestad et al., 2017
	RvD2			Botten et al., 2019
	RvE1			Lippestad et al., 2017
	Mar1		Increases glycoconjugate secretion Counter-regulates histamine-induced secretion	Olsen et al., 2020
	RvD1 & RvE1	Sprague-Dawley Rat and Human primary conjunctival goblet cells	Inhibits leukotriene-induced glycoconjugate secretion	Dartt et al., 2011
	RvD1 & AT-RvD1		Counter-regulates histamine-induced secretion	Li et al., 2013
***In vivo* studies**				
**Asthma**	RvE1	BALB/c mice	Lowers mucus score	Aoki et al., 2008

**Figure 5:**
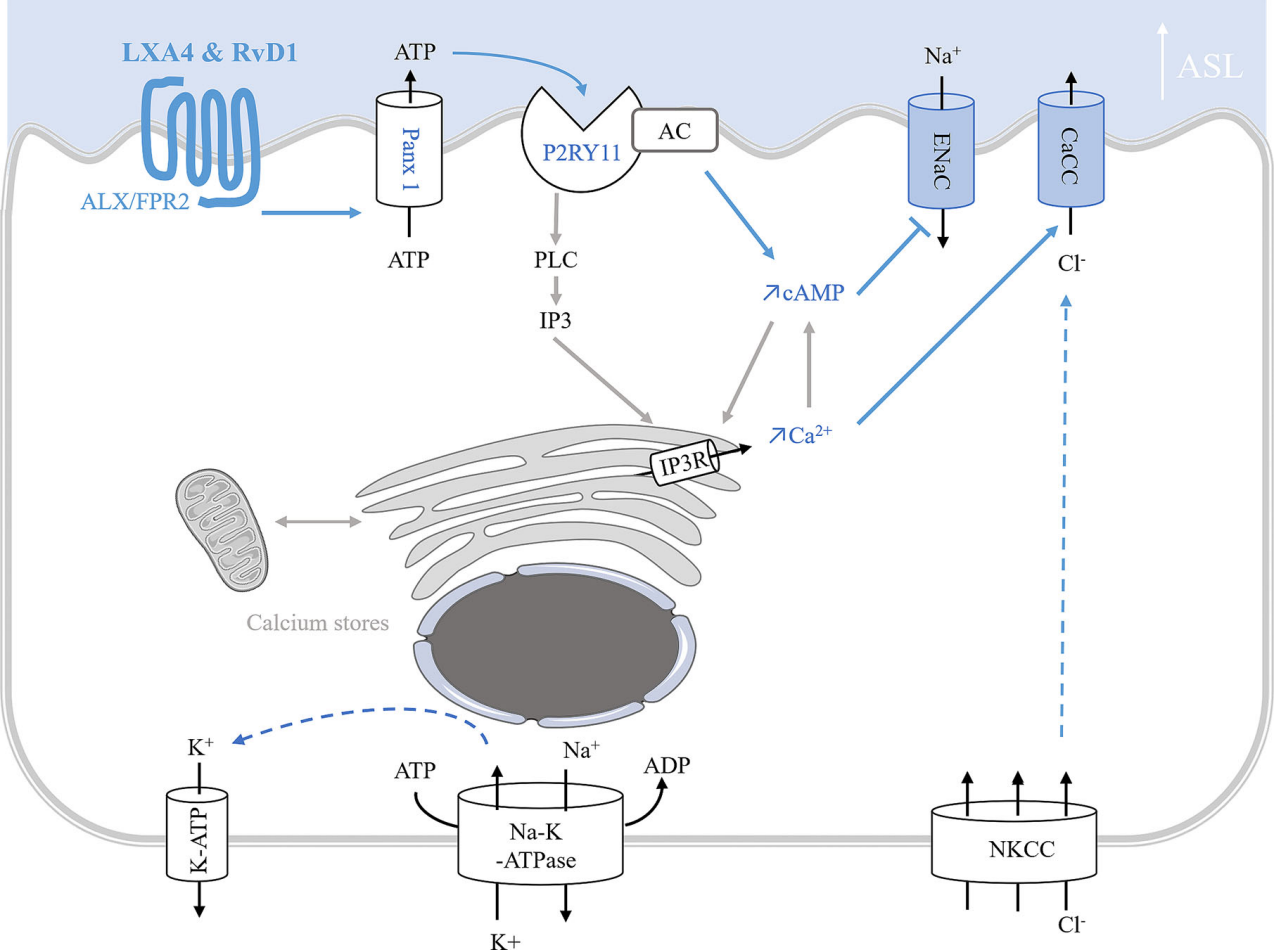
Model of ion transport regulation by LXA4 and RvD1 enabling the restoration of ASL height in CF human bronchial epithelial cells. LXA4 and RvD1 induce a calcium-dependent airway surface liquid (ASL) layer height increase in human CF bronchial epithelial cell lines and primary cultures (Bonnans et al., 2003; Al-Alawi et al., 2014; Higgins et al., 2014; Ringholz et al., 2018; Philippe and Urbach, 2018). This effect involves binding to the FPR2 receptor, stimulation of an ATP secretion into the airway lumen via pannexin 1 channels (Panx1), and subsequent activation of P2Yx purino-receptors, including P2Y11 which leads to a rise in both intracellular cAMP and calcium mobilization by stimulation of adenylate cyclase (AC) activity and inositol 3 phosphate (IP3) signaling pathway. Intracellular calcium and cAMP increases induce stimulation of calcium-activated chloride channel and inhibition of ENaC channel inhibition.

*In vitro studies of ion transport in CF airway epithelial cells*. In human CF bronchial epithelial cells, LXA4 and RvD1 stimulate an increase of the ASL to a normal height (7 µm) (Verrière et al., 2012; Al-Alawi et al., 2014; Higgins et al., 2014; Ringholz et al., 2018). Stimulation of the surface airway hydration by these SPMs can be inhibited by the FPR2 receptor antagonist, BOC-2 and by BAPTA-AM, indicating this process to be mediated by the FPR2 receptor and highly dependent on intracellular calcium. Furthermore, LXA4 induces a large and sustained intracellular calcium increase in CF airway epithelial cells involving the stimulation of an apical ATP secretion by pannexin channels (PANX1). The subsequent activation of purino-receptors localized in these cells’ apical membrane generate calcium entry and calcium release from intracellular stores. A role for the P2Y11, a GPCR purino-receptor leading to a rise in both intracellular cAMP (stimulation of adenylate cyclase [AC]) and calcium mobilization (increased inositol 3 phosphate [IP3]) has been demonstrated upon exposure of CF airway epithelia cells to LXA4 (**Figure 5**) (Bonnans et al., 2003; Verrière et al., 2012; Higgins et al., 2014). In human CF airway epithelial cells, patch-clamp studies have demonstrated that LXA4 stimulates a calcium-activated chloride secretion (Verrière et al., 2012). Another study reported that the LXA4 effect in restoring the ASL height in CF airway epithelial cells also implicates ENaC inhibition (Al-Alawi et al., 2014) (**Figure 5**, **Table 1**).

In vivo *studies of ion transport in CF airway epithelial cells*. The role of LXA4 and RvD1 on chloride secretion and sodium absorption was further confirmed *in vivo* by the recovery of nasal transepithelial potential difference in homozygous F508del CFTR mice (Ringholz et al., 2018) (**Table 1**).

### Cytokine Production and Release

**Figure 6:**
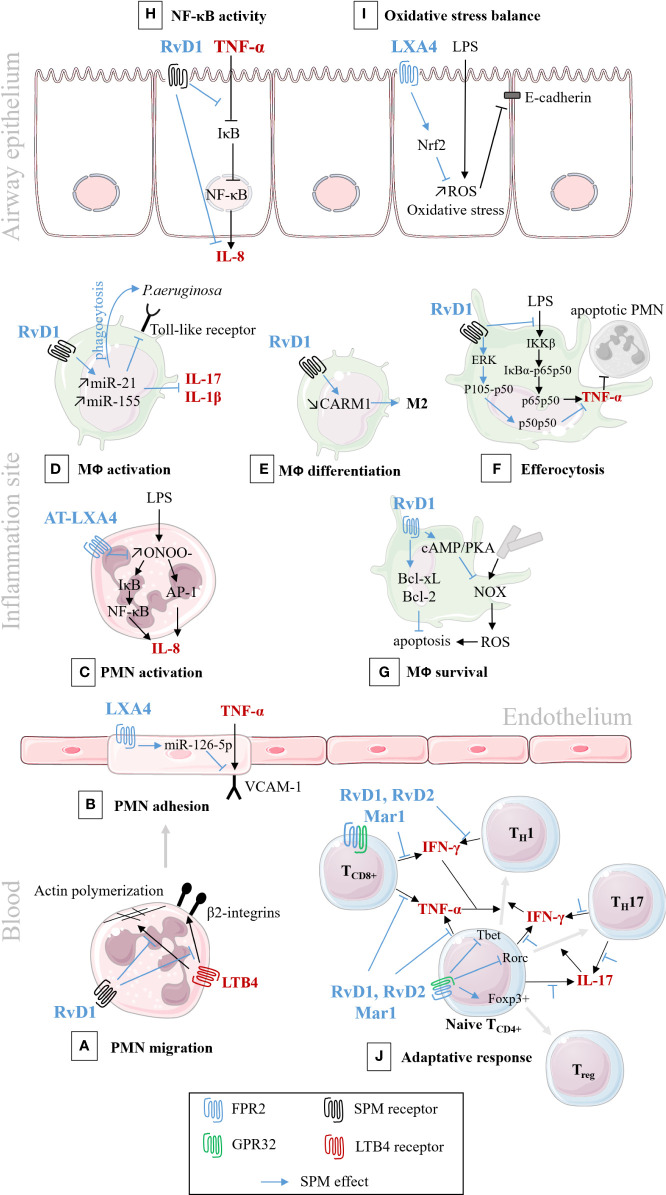
Schematic illustration of SPMs cellular pathways involved in the resolution of inflammation/infection. In black: pro-inflammatory pathways; In blue: bioactions of SPM. **(A)** RvD1 analog down-regulates the migration of PMNs isolated from human peripheral blood, by decreasing actin polymerization, and blocking LTB4-regulated adhesion molecules, 2 integrins. (Krishnamoorthy et al., 2010); **(B)** LXA4 down-regulates VCAM-1 receptor and PMN adhesion to human endothelial cells (Codagnone et al., 2017); **(C)** AT-LXA4 (15-epi-LXA4) down-regulates IL-8 synthesis and secretion, in human PMNs isolated from venous blood (Jozsef et al., 2002); **(D)** RvD1 down-regulates toll-like receptor (TLR), cytokines synthesis and enhances phagocytosis of murine lung macrophage (Codagnone et al., 2018); and enhances phagocytosis and bacterial killing in human CF alveolar macrophage (Ringholz et al., 2018); **(E)** RvD1 stimulates murine macrophage differentiation from M1 to M2 state (Recchiuti et al., 2014); **(F)** RvD1 stimulates efferocytosis of apoptotic PMNs, through the regulation of NF-κB pathways, in murine macrophage (Lee et al., 2013); **(G)** RvD1 inhibits ROS production and increases murine macrophage survival after efferocytosis (Lee and Surh, 2013); **(H)** RvD1 inhibits cytokines secretion by preventing IκB degradation in CF human airway epithelial cell (Ringholz et al., 2018); **(I)** LXA4 inhibits oxidative stress and protects E-cadherin, in human airway epithelial cell (Cheng et al., 2016); **(J)** RvD1, RvD2, and Mar1 regulate the adaptive response, enhance the differentiation to T_reg_ rather than T_H_1 and T_H_17, in human peripheral blood lymphocytes (Chiurchiù et al., 2016).

As part of the inflammatory response, the role of SPMs in inhibiting pro-inflammatory cytokines production (e.g.: IL-6, IL-8, IL-1β, and TNFα, IL-13) and stimulating anti-inflammatory cytokines (IL-4, IL-10) as well as inhibiting leukocyte chemotaxis and migration has been demonstrated in many chronic diseases. Their role has been established in models of acute lung injury, asthma, and COPD (Lee et al., 1989; Serhan et al., 1995; Papayianni et al., 1996; Hachicha et al., 1999; Bonnans et al., 2002; Vachier et al., 2005; Bonnans et al., 2006; Sun et al., 2007; Aoki et al., 2008; Isobe et al., 2012; Yang et al., 2013; Croasdell et al., 2015; Codagnone et al., 2018) as well as cellular models of viral and bacterial inflammation, or oxidative stress (Chiang et al., 2012; Hsiao et al., 2014; Wang L. et al., 2014; Wang Q. et al., 2014; Cox et al., 2015) and cigarette smoke-induced inflammation (Takamiya et al., 2012; Jiajia et al., 2014) (**Table 3**). The pathways involved in SPM’s regulation of inflammation are presented in **Figure 6**.

**Table 3:** Bioactions of SPMs on cytokine secretion and leukocytes activity, demonstrated in non-CF models.

Cytokines synthesis /Leukocytes activity				
Conditions	SPMs	Models	Bioactions	References
***In vitro* studies**				
**TNF-α treatment**	LXA4	Human umbilical vein endothelial cells	Up-regulates miR-126-5p (related to endothelial repair and VCAM-1 expression)	Codagnone et al., 2017
	LXA4 and AT-LXA4 analog	Human peripheral blood PMNs	Inhibit TNF-α-initiated neutrophil migration, superoxide generation and IL-1β release	Hachicha et al., 1999
**TGF-β1-induced fibrosis**	LXA4	Human renal epithelial HK-2 cell line Human mesangial cells Rat renal fibroblast cells (NRK49F)	Up-regulates miR let-7c (reduces renal fibrosis) in human cells Decreases miR let-7c level in rat cells	Brennan et al., 2013
**Intestinal *Salmonella Typhimurium***	LXA4 analog	Human intestinal epithelium T84 cell line	Inhibits IL-8 secretion	Gewirtz et al., 1998
**Airway *P. aeruginosa***	AT-LXA4 analog	Human primary bronchial epithelial cells	Inhibits IL-8 secretion	Karp et al., 2004
**Chemoattractant**	LXA4	Human peripheral blood PMNs	Inhibits LTB4/FMLP-induced PMN chemotaxis and migration	Lee et al., 1989
	LXA4, LXB4	Human peripheral blood PMNs, HUVEC	Inhibit LTB4-induced PMN adhesion to endothelial cells and migration across endothelial cell monolayers	Papayianni et al., 1996
	LXA4 analog	Human peripheral blood PMNs, HL-60 cells, HUVEC	Inhibit PMN transendothelial migration and endothelial adhesion	Serhan et al., 1995
	RvD1, AT-RvD1	Human peripheral blood PMNs and microvascular endothelial cells (HMEC-1)	Inhibit PMN transendothelial migration	Sun et al., 2007
	RvE3	Human peripheral blood PMNs	Inhibits PMN chemotaxis	Isobe et al., 2012
**Acid lung injury**	LXA4	Human bronchial epithelial (HBE) and type II alveolar (A549) cell lines	Inhibits IL-6 release and PMN transendothelial migration	Bonnans et al., 2006
**Asthma**	LXA4, LXB4	Human asthmatic peripheral blood mononuclear cells	Inhibit IL-8 release	Bonnans et al., 2002
**Cigarette smoke**	RvD1, RvD2	Human peripheral blood monocytes and alveolar macrophages	Decrease release of pro-inflammatory cytokines (IL-6, IL-8 and TNF-α) and increase release of IL-10 and TGF-β Stimulate phagocytosis and M2 state Decrease levels of protein carbonylation after 24h	Croasdell et al., 2015
	RvE1	Murine macrophage RAW264.7cell line	Stimulates phagocytic activity and reduces cell death	Takamiya et al., 2012
	RvD1	Human bronchial epithelial 16HBE cell line	Reduces IL8 synthesis	Jiajia et al., 2014
	AT-RvD1	Human THP1 macrophages and alveolar epithelial A549 cell lines	Reduces IL-1β secretion in macrophages and IL-6 and IL-8 secretion in alveolar cells	Cox et al., 2015
**Polyinosinic-polycytidylic acid**	RvD1	Human primary lung epithelial cells	Inhibits IL-6 and IL-8 release	Hsiao et al., 2014
**Microbial sepsis**	RvD2 analog	Human primary PMNs and HUVEC	Stimulates NO production in HUVEC Reduces L-selectin and CD-18 surface expression Stimulates phagocytosis of *E. Coli* Increases intracellular ROS	Spite et al., 2009
***E. Coli***	RvD1 analog, RvD5 analog, PD1	Human peripheral blood monocyte and PMN	Increase phagocytosis of *E. Coli* (RvD1,RvD5, PD1) Regulate inflammatory genes (RvD1, RvD5)	Chiang et al., 2012
	Mar1, 22-OH-MaR1, 14-oxo-MaR1	Human primary peripheral blood macrophages	Increase macrophage phagocytosis	Colas et al., 2016
**PMA/Ionomycin**	RvD1, RvD2, AT-RvD3 MaR1	Human peripheral blood CD8+ and CD4+ T cells	Inhibit TNF-α, IFN-γ, IL-2 production Favor CD+4 T cells differentiation into regulatory T cells Inhibit naive CD+4 T cells differentiation into TH1 and TH17 cells	Chiurchiù et al., 2016
**Other**	RvE1, RvD1, RVD2, PD1	Human peripheral blood mononuclear cells	RvD1 regulates miR-146b, miR-21, miR-208 and miR-219-5p RvE1 regulates miR-219-5p and miR-21 but not miR146b RvD2 and PD1 regulate miR-146b and miR-21 but not miR-219-5p	Fredman et al., 2012
	PDn-3 DPA	Human peripheral blood macrophages	Regulates monocyte-to-macrophage differentiation	Pistorius et al., 2018
**Sickle-cell disease (SCD)**	AT-RvD1 and RvD1	Human endothelial cell line, human SCD blood PMN and erythrocytes	Inhibit neutrophil recruitment Stimulate erythrocytes and PMN efferocytosis	Matte et al., 2019
***In vivo* studies**				
**TNF-α**	LXA4 and AT-LXA4 analog	BALB/c mice	Inhibit leukocyte infiltration	Hachicha et al., 1999
**Chronic renal fibrosis**	LXA4	Male wistar rats	Up-regulates miR let-7c (reduces renal fibrosis)	Brennan et al., 2013
**LPS-induced lung injury**	LXA4, RvD1	Male Sprague-Dawley rats (BAL)	Reduce TNF-α, IL-6 and IL-1β secretion Increase IL-10 secretion	Wang Q. et al., 2014, Yang et al., 2013
**Acute peritonitis**	RvD1, AT-RvD1	Male FVB mice	Reduce leukocyte infiltration	Sun et al., 2007
	RvD1	C57Bl/6N mice	Reduces leukocytes infiltration Stimulates M2 differentiation and activity Regulates corresponding genes	Recchiuti et al., 2014
	RvD1 analog	Male FVB mice	Temporally controls miRNAs: at 12h, increases miR21 and miR146b, decreases miR208a (NF-kB signaling) and miR219 (LTB4 production)	Recchiuti et al., 2011
	RvE3	Male FVB mice	Inhibits PMN infiltration	Isobe et al., 2012
**Asthma**	RvE1	Female BALB/c mice	Lowers mucus score	Aoki et al., 2008
**Microbial sepsis**	RvD2 analog	Male FVB mice, eNOS-/- & wild type (C57BL/6J) mice	Reduces neutrophils recruitment, leukocyte-endothelial interactions (NO-dependent) Reduces local and systemic bacterial burden Increases peritoneal mononuclear cells Increases survival Reduces IL-6, IL-1β, IL-23, TNF-α	Spite et al., 2009
**Peritoneal *E. Coli* & Skin *S. aureus***	RvD1 analog, RvD5 analog, PD1	Male FVB mice, C57BL/6J male mice	Reduces bacterial burden in blood and exudates (RvD1, RvD5) Increase survival (RvD1, RvD5) Amplify antimicrobial response of ciprofloxacin Increase clearance of *S. aureus* with vancomycin Reduce IL-1β, IL-6 and increase IL-10 secretion (RvD1) Reduce KC and TNF-α secretion (RvD5)	Chiang et al., 2012
**Peritoneal *E. Coli***	PDn-3 DPA	Male C57Bl/6N mice	Increases macrophages' efferocytosis and bacterial phagocytosis	Pistorius et al., 2018
**Sickle-cell disease (SCD)**	AT-RvD1	SCD and healthy (Hba^tm1(HBA)Tow^ Hbb^tm3(HBG1, HBB)Tow^) mice	Reduces neutrophils adhesion & transmigration Reduces lung and kidney injury Reduces proinflammatory cytokine	Matte et al., 2019

In vitro *studies in CF*. In CF airway epithelial cells, LXA4 and RvD1 inhibit IL-8 synthesis by preventing IκB degradation induced by TNFα, thus resulting in NF-κB (Ringholz et al., 2018). RvD1 regulates specific genes and proteins involved in leukocyte chemotaxis and infiltration (e.g. CXCL1 [the mice IL-8]) in lung macrophages isolated from mice infected with *P. aeruginosa* (Codagnone et al., 2018; Isopi et al., 2020) (**Table 1**).

*In vivo* studies in *P. aeruginosa infected mice*. AT-LXA4 stable analog, reduces IL-8 levels and PMN recruitment in a murine model of short-term *P. aeruginosa* lung infection (Karp et al., 2004). RvD1 treatment also produces a significant decrease in PMN, IL-1β, and CXCL1 levels *in vivo* in the lungs of mice infected with *P. aeruginosa* (Codagnone et al., 2018) (**Table 1**).

*Human studies in CF*. Although, not a direct demonstration of SPMs’ impact on cytokine secretion, it is worth noting that human studies reveal that the levels of pro-inflammatory cytokines (IL-6 and IL-8) are inversely correlated with LXA4 and RvD1 in the sputum of patients with CF (Chiron et al., 2008; Ringholz et al., 2014; Eickmeier et al., 2017; Isopi et al., 2020).

### Infection

Many actions exerted by SPMs to limit infection have been described in non-CF cells isolated from human and mice macrophages, (Chiang et al., 2012; Colas et al., 2016; Pierdomenico et al., 2017; Codagnone et al., 2018) (**Table 3**). In addition, SPMs (PDn-3DPA, RvD1, and RvD2) stimulate macrophage differentiation from a pro-inflammatory (M1) to a pro-resolutive phenotype (M2) in non-CF models (Dalli and Serhan, 2012; Recchiuti et al., 2014; Croasdell et al., 2015; Pistorius et al., 2018) and enhance the expression of surface receptors involved in the uptake of apoptotic cells (Matte et al., 2019) (**Table 3**). RvD2 also reduces polymicrobial sepsis severity in mice (Spite et al., 2009) and MaR1 and RvD3 enhance *E. coli* phagocytosis by macrophages (Colas et al., 2016) (**Table 3**).

In vitro *studies in CF model of infection*. The role of SPMs in regulating infection was also demonstrated in CF. RvD1 enhances phagocytosis of *P. aeruginosa* by human CF alveolar macrophages (Ringholz et al., 2018). LXA4 also stimulates CF bronchial epithelial cells’ protective functions by delaying *P. aeruginosa* invasion and migration (Higgins et al., 2016) (**Table 1**).

In vivo *studies in CF model of infection*. During *P. aeruginosa* lung infection in mice, exogenous administration of SPMs significantly decreases bacterial load, and improves clinical outcome. An AT-LXA4 stable analog, reduces bacterial burden in a murine model of short-term *P. aeruginosa* lung infection (Karp et al., 2004). RvD1 significantly diminishes bacterial growth, mucus metaplasia and lung inflammation resulting from long term exposure of mice to *P. aeruginosa* (Codagnone et al., 2018) (**Table 1**).

### Epithelial Repair

**Figure 7:**
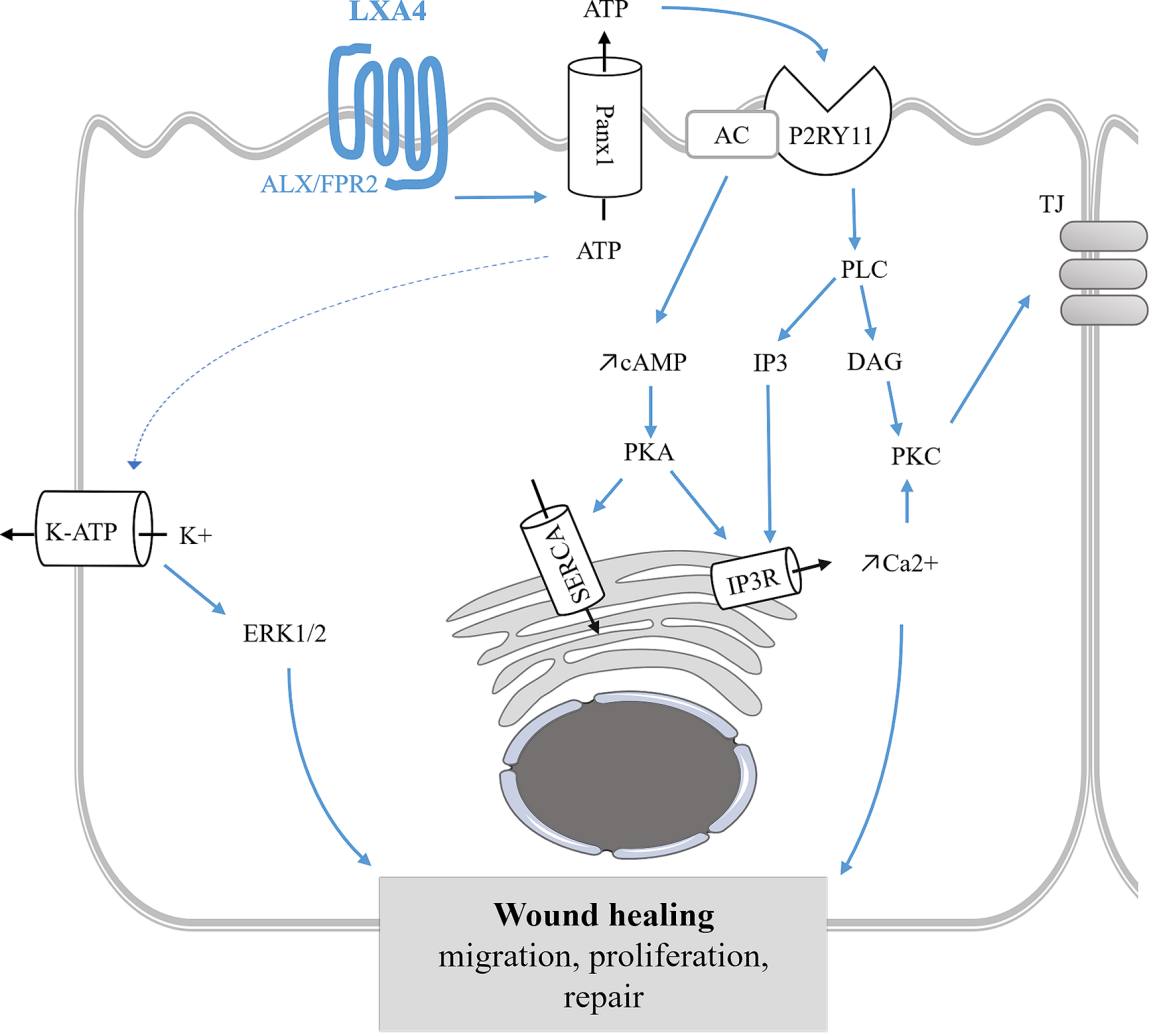
Epithelial repair mediated by LXA4 in bronchial epithelium. LXA4 stimulates wound healing by enhancing cell proliferation, migration of human CF bronchial epithelial cells. This mechanism is mediated by the FPR2 receptor, an apical ATP release and the subsequent stimulation of P2Yx receptors leading to intracellular calcium mobilization. The CF airway epithelial repair induced by LXA4 also involves ERK1/2 phosphorylation and KATP channel stimulation (Buchanan et al., 2013; Higgins et al., 2014). LXA4 maintains airway epithelial structure by stimulating tight junction (TJ) proteins synthesis and trafficking to the apical membrane leading to an increase of transepithelial electrical resistance (Grumbach et al., 2009). SPMs stimulation of the ERK pathway has also been demonstrated in corneal epithelial repair (Xu et al., 2009; Zhang et al., 2010; Kenchegowda et al., 2011; Zhang et al., 2018).

*In vitro* studies have shown that SPMs (LXA4, RvD1, AT-RvD1 RvD2, and RvE1) protect tissues from lung injury in numerous models including CF airway epithelium (Zhang et al., 2010; Kenchegowda et al., 2011; Kakazu et al., 2012; Odusanwo et al., 2012; Buchanan et al., 2013; Higgins et al., 2014: Cheng et al., 2016; Higgins et al., 2016; Zhang et al., 2018; Zheng et al., 2018). LXA4 stimulated wound healing by enhancing cell proliferation, migration in human CF bronchial epithelial cells (Buchanan et al., 2013; Higgins et al., 2014) through stimulation of the ALX/FPR2 receptor, intracellular calcium mobilization, KATP potassium channel activation, and the mitogen-activated protein kinase ERK1/2 phosphorylation (Buchanan et al., 2013; Higgins et al., 2014). Moreover, LXA4 restores transepithelial resistance in CF airway epithelial cells by enhancing tight junction formation *via* upregulation of expression of the proteins ZO-1, occludin, claudin-1 during bacterial infection by *P. aeruginosa* (Grumbach et al., 2009; Higgins et al., 2016) (**Figure 7**) (**Table 1**). In corneal epithelium, the role of mitogen-activated protein kinase has also been reported to mediate the epithelial repair induced SPMs. The serine/threonine kinase Akt and NrF2 signaling pathways have been described in mediating the response to SPMs in corneal and other epithelial models (Zhang et al., 2010; Kenchegowda et al., 2011; Odusanwo et al., 2012; Wang L. et al., 2014; Posso et al., 2018; Zhang et al., 2018) (**Table 4**).

**Table 4:** Bioactions of SPMs on epithelial repair and oxidative stress, demonstrated in non-CF models.

Epithelial repair/Oxidative stress				
Conditions	SPMs	Models	Bioactions	References
***In vitro* studies**				
**Corneal injury**	LXA4	Rabbit corneal epithelial cells	Increases cell proliferation and migration	Kenchegowda et al., 2011
	RvE1	Human corneal epithelial cell line	Stimulates migration	Zhang et al., 2010
**Acute lung injury**	LXA4	Human bronchial epithelial 16HBE cell line	Protects airway epithelial through inhibition of LPS-induced ROS production. Preserves E-cadherin expression	Cheng et al., 2016
	RvD1	Human primary alveolar type II epithelial cell Human primary lung fibroblast	Enhances cell wound repair and proliferation, differentiation. Reduces apoptosis. Inhibits TGF-Beta-induced epithelial-mesenchymal transition. Inhibits fibroproliferation and myofibroblast differentiation of human lung fibroblasts	Zheng et al., 2018
**TNF-α treatment**	RvD1	Rat parotid Par-C10 cell line	Inhibits tight junction and cytoskeletal disruption Stimulates cell migration and polarity	Odusanwo et al., 2012
**Cigarette smoke**	RvD1, RvD2	Human primary peripheral blood monocytes and macrophages, and alveolar macrophages	Decrease levels of protein carbonylation after 24h	Croasdell et al., 2015
	RvD1	Human bronchial epithelial 16HBE cell line	Reduces the production of extracellular H2O2	Jiajia et al., 2014
	AT-RvD1	Male C57BL/6 mice (lung)	Reduces oxidative stress markers and ROS in BAL Restores elastic fibers Reduces alveolar enlargement	Posso et al., 2018
**Microbial sepsis**	RvD2 analog	Primary human PMNs; primary human umbilical vein endothelial cell *(HUVEC)*	Inhibits extracellular superoxide generation	Spite et al., 2009
**Other**	LXA4	Human bronchial epithelial 16HBE14o- cell line	Stimulates formation of tight junction through ZO-1, occludin, claudin-1 expression	Grumbach et al., 2009
	RvD1	Murine macrophages RAW264.7 cell line	Inactivates NADPH oxidase (NOX). Increases anti-apoptotic proteins	Lee and Surh, 2013
***In vivo* studies**				
**Corneal injury**	LXA4	C57BL/6 mice	Reduces surface area of injury	Kakazu et al., 2012
	RvD1	Diabetic C57BL/6 mice	Stimulates regeneration of the corneal epithelium Diminishes ROS accumulation by increasing GSH synthesis and decreasing NOX expression	Zhang et al., 2018
**Acute lung injury**	LXA4	BALB/c mice	Protects airway epithelial through inhibition of LPS-induced ROS production. Preserves E-cadherin expression	Cheng et al., 2016
	RvD1		Reduces production of lipid peroxidation malondialdehyde. Increases expression of SOD and HO-1 mRNA	Wang Q. et al., 2014

## Other Possible Impacts of SPMs on Altered Airway Functions in CF

Although not demonstrated in CF, the role of SPMs on airway functions altered in CF but demonstrated in other disease models can be pointed out (**Tables 2**–**5**).

**Table 5:** Bioactions of SPMs on micro-RNA regulation, demonstrated in non-CF models.

micro-RNA				
Conditions	SPMs	Models	Bioactions	References
***In vitro* studies**				
**TNF-α treatment**	LXA4	Human umbilical vein endothelial cells	Up-regulates miR-126-5p (related to endothelial repair and VCAM-1 expression)	Codagnone et al., 2017
**TGF-β-induced fibrosis**	LXA4	Human renal epithelial HK-2 cells Human mesangial cells Rat renal NRK49F fibroblasts	Up-regulates miR let-7c and reduces human renal fibrosis Decreases miR let-7c level in rat fibroblasts	Brennan et al., 2013
**Other**	RvD1 RvE1 RVD2 & PD1	Human peripheral blood mononuclear cells	Regulates miR-146b, miR-21, miR-208 and miR-219-5p Regulates miR-219-5p and miR-21 but not miR146b Regulate miR-146b and miR-21 but not miR-219-5p	Fredman et al., 2012
***In vivo* studies**				
**Chronic renal fibrosis**	LXA4	Male wistar rats	Up-regulates miR let-7c and reduces renal fibrosis	Brennan et al., 2013
**Acute peritonitis**	RvD1 analog	Male FVB mice	Temporally controls miRNAs, increases miR21 and miR146b, decreases miR208a (NF-kB signaling) and miR219 (LTB4 production)	Recchiuti et al., 2011

### Oxidative Stress

Antibiotics, despite their beneficial action on pulmonary function during acute infections, do not treat oxidative stress (Wood et al., 2002). Although not demonstrated in CF, SPMs have been found to help reduce oxidative stress by restoring oxidant/antioxidant balance through the nuclear erythroid 2-related transcription factor 2 (Nrf2) pathway, which is defective in CF (Posso et al., 2018; Zhang et al., 2018). In the murine model of cigarette smoke- and LPS-induced lung injury, or type 1-diabetes, RvD1 reduces ROS levels and increases antioxidant defense (Wang L. et al., 2014; Posso et al., 2018; Zhang et al., 2018). In corneal epithelium of type 1-diabetic mice, RvD1 increases the amount of the antioxidant GSH (Zhang et al., 2018). *In vitro*, following induced-efferocytosis, RvD1 inactivates NOX in murine macrophages (Lee and Surh, 2013). In human macrophages and bronchial epithelial cells undergoing cigarette-induced oxidative stress, RvD1 and RvD2 decrease protein oxidation (Croasdell et al., 2015) and decrease extracellular H_2_O_2_ production (Jiajia et al., 2014) (**Table 4**).

### Mucus Secretion

Recent studies reveal the impact of SPMs on mucus secretion. Although not studied in CF, RvD1, LXA4, and Mar1 mediated an increase in glycoconjugate and mucin secretion in conjunctival cells (Hodges et al., 2017; Lippestad et al., 2017; Olsen et al., 2020). However, Mar1, RvD1, RvE1, and AT-RvD1 are able to block conjunctival goblet cell secretion when triggered by histamine or leukotriene (Dartt et al., 2011; Li et al., 2013; Olsen et al., 2020). In asthma, RvE1 reduced mucus score (Aoki et al., 2008) and RvD1 lowered mucous metaplasia in infected mice (Codagnone et al., 2018) (**Table 2**).

### Regulation of MicroRNA

The microRNA signature of resolution of inflammation has recently started to be reported. Studies at basal state or during inflammation in mice and human leukocytes have revealed the capacity of resolvins (RvD1/D2, RvD1 stable analog, PD1 and RvE1) to regulate miRNAs that influence NF-κB activity and cytokine production (miR21, miR146b, miR208a, miR155), endothelial integrity (miR-126), leukotriene production (miR219), and pathogen recognition (miR21) (Recchiuti et al., 2011; Fredman et al., 2012; Codagnone et al., 2017; Codagnone et al., 2018). LXA4 also attenuates renal fibrosis by inducing let-7c (Brennan et al., 2013). Furthermore, miR-181b which is overexpressed in CF macrophages and airway epithelial cells, down-regulates the expression of the FPR2 receptor and the pro-resolution signaling pathways (Pierdomenico et al., 2015; Pierdomenico et al., 2017) (**Table 5**).

### Adaptative Immunity

SPMs might also play a role in the mediation of the adaptive response. RvD1, RvD2, and Mar1 modulate adaptive immune responses in human peripheral blood lymphocytes. These SPMs prevent naïve CD4^+^ T cell differentiation into T_H_1 and T_H_17 in a mechanism mediated by the GPR32 and the FPR2 receptors (Chiurchiù et al., 2016). This role of SPMs in adaptive immunity might be of interest for CF. Indeed, some reports suggested T lymphocytes and activated eosinophils in airway mucosa in CF (Azzawi et al., 2012) and intrinsic impairment of T cell differentiation may contribute to the greater severity and more rapid progression of CF lung disease (Kushwah et al., 2014).

## CF Airway Disease Treatments and Inflammation

### Antibiotics

Until recently, antibiotics have been the only therapeutic agents that could be used to treat airways inflammation in CF. Antibiotics can work in synergy with the host immunity to kill bacteria with great efficiency. Antibiotics influence immune responses in various ways beyond merely eliminating the source of inflammation, depending on how they are used and how effective they are. Indeed, antibiotics that induce bacteria lysis can lead to the release of highly inflammatory molecules, pathogen-associated molecular patterns (PAMPs), such as lipopolysaccharide (LPS) and therefore augment inflammation (Raetz and Whitfield, 2002).

In contrast, some antibiotics have shown immunomodulatory properties (Ruh et al., 2017) such as macrolides which suppress inflammatory cytokines (Hoyt and Robbins, 2001; Amsden, 2005). Azithromycin has been particularly studied in CF and is routinely used for its immunomodulating effects, having shown to reduce exacerbation frequency and slightly improve lung function when taken continuously (Bush and Rubin, 2003; Southern and Barker, 2004; Olveira et al., 2017). Of interest, while PMN counts and IL-8 levels decrease in CF patients’ sputum after antibiotic therapy, LXA4 levels significantly increase and are inversely correlated with IL-8 levels (Chiron et al., 2008). However, despite its beneficial effects in CF patients, said effects are marginal, especially when taking into account the list of oral treatments patients already contend with on a daily basis, without mentioning the long half-life of this antibiotic and the potential risk of selecting resistant bacteria strains and altering microbiota.

### CFTR Modulators

High-throughput screening has allowed to identify small molecules, such as lumacaftor (VX809) tezacaftor (VX661), and elexacaftor (VX-445) to modulate processing and trafficking of CFTR (CFTR correctors) and ivacaftor (VX770) to potentiate its activity as a chloride channel (CFTR potentiator) providing the first drugs to specifically target the CFTR protein defect. Associations of these molecules, the first ones being currently prescribed in routine since the mid-2010s, restore chloride transport and normalizing sweat test results (Lopes-Pacheco, 2019). Beside their main action on epithelial chloride transport and encouraging results on disease progression, their impact on inflammatory cytokine production are controversial and their long term immunomodulatory effects are still discussed (Jarosz-Griffiths et al., 2020; Volkova et al., 2020). Indeed, the corrector VX809 does not produce an effect on proinflammatory cytokines production by macrophages exposed to *P. aeruginosa* while stimulating their phagocytosis activity. In contrast, the potentiator VX770 reduces proinflammatory cytokines but inhibits macrophages’ phagocytosis activity (Barnaby et al., 2018). VX809 alone or in combination with VX770 does not affect pro-inflammatory cytokines production by CF airway epithelial cells. *P. aeruginosa* exposure however reduces VX809-stimulated F508del-CFTR chloride secretion by airway epithelial cells (Stanton et al., 2015). However, another study has shown that correcting CFTR activity by a combination of VX809 and VX770 reduces IL-8 production by CF (F508del) airway epithelial cells and enhanced epithelial repair (Ruffin et al., 2018). The effects of the latest VX-445 on inflammation have yet to be assessed. Although these small molecules have considerably changed the quality of life for many patients, not all CF genotypes can benefit from such therapy. Moreover, the clinical response within an identical genotype is variable and lung function still declines over time when bronchiectasis is established.

### Novel Anti-Inflammatory Molecules

To combat inflammation, non-steroid anti-inflammatory drugs (NSAID) have been studied; especially Ibuprofen, which seems to slow lung disease progression (Lands and Stanojevic, 2013) but at the cost of long term usage at high doses and the entailing risks.

Taking another approach and directly targeting biosynthesis of the inflammatory eicosanoid LTB4, acebilustat is a new anti-inflammatory oral drug that inhibits LTA4H. As LTB4 is one of the inflammatory eicosanoids that initiate and amplify PMNs’ recruitment, suppressing its effect could diminish the pulmonary consequences of overactive PMNs. In a phase I study, acebilustat has decreased PMN inflammation biomarkers levels in sputum and was well tolerated with only mild to moderate adverse events reported (Elborn et al., 2017). However, a phase II randomized control trial (RCT) did not meet its primary endpoint and no further trials with acebilustat are planned (Elborn et al., 2018). In addition, these anti-inflammatory molecules clearly have a different mechanism of action than immuno-resolvents.

Lenabasum, formerly called JBT-101, is another oral drug under investigation for autoimmune diseases and CF. A first phase II trial conducted with 83 patients has established the safety and tolerance of this drug in CF patients. Another phase II multi-center RCT is ongoing for CF patients with pulmonary exacerbations as the primary outcome. Lenabasum is an endocannabinoid-mimetic that selectively binds to cannabinoid receptor 2 expressed by immune cells, reducing alveolar macrophage secretion of IL-6 and TNFα in preclinical studies (Ribeiro et al., 2017). Those immunomodulatory effects can be explained by lenabasum’s potential to induce SPMs production in animal models (Zurier et al., 2009) and healthy human subjects (Motwani et al., 2018a). This last example showcases how treating chronic inflammation by mobilizing the body’s endogenous pro-resolving system rather than trying to stop one of the many triggers of an established and thriving inflammatory process may be the most inclusive and efficient way to treat inflammatory diseases in the future.

## Conclusion

Nearly 30 years after the discovery of the CFTR gene, many aspects of the pathophysiology of CF remain unclear. The combination of advanced knowledge in the field of the regulation of innate immunity and the current description of CF airway disease revealed novel cellular and molecular mechanisms involved in the abnormal resolution of inflammation in CF. This review highlighted evidence for a role of SPMs to overcome the absence of functional CFTR by stimulating chloride secretion through calcium-dependent channels, while limiting unwanted persistence of inflammation, infection, and tissue damage, and accelerating the return to homeostasis by acting on multiple cells and molecular targets (**Figure 4**).

Even in the age of CFTR modulators, anti-inflammatory therapy remains an area of intense research in CF. The therapeutic use of SPMs might constitute a promising avenue to treat chronic inflammation and infection in all CF patients. However, the therapeutic use of SPMs is greatly limited by their rapid metabolic inactivation before they can reach the site of inflammation. An RvE1 stable analog for dry eye inflammation (NCT00799552), ocular inflammation and pain in cataract surgery (NCT02329743), and allergic conjunctivitis (NCT01639846), and a LXA4 stable analog for periodontal inflammation (NCT0234269) are under clinical trials. The use of SPMs stable analog incorporated in nanoparticles also provides a new possibility for local delivery at the site of inflammation (Van Dyke et al., 2015; Lance et al., 2017). Although promising results have been obtained in animal studies, the efficacy of this experimental data must be established in human clinical trials. Indeed, using analogs with a much longer half-life than native SPMs does raise the question of the potential adverse effects. Long-term exposure to stable SPM analogs with prolonged and enhanced activity might prevent the initiation of further inflammatory responses. Another approach would be to understand more precisely the mechanism by which CFTR dysfunction is related to an abnormal SPMs biosynthesis and potentially reveal new therapeutic targets to treat a wide range of CF mutations. Furthermore, the presentation of CF is very heterogeneous in terms of severity and response to actual treatments; therefore, exploring the correlation at the individual level between SPMs biosynthesis, CF genotype, and the severity of the airway disease could lead to new diagnostic and therapeutic tools as personalized care.

## Author Contributions

MB wrote the first draft on the impact of SPMs on airway functions altered in CF. MS wrote the first draft of the abnormal lipid metabolism and SPMs biosynthesis in CF. All authors contributed to the article and approved the submitted version.

## Conflict of Interest

The authors declare that the research was conducted in the absence of any commercial or financial relationships that could be construed as a potential conflict of interest.
